# Evaluating the feasibility of AI-predicted bpMRI image features for predicting prostate cancer aggressiveness: a multi-center study

**DOI:** 10.1186/s13244-024-01865-8

**Published:** 2025-01-15

**Authors:** Kexin Wang, Ning Luo, Zhaonan Sun, Xiangpeng Zhao, Lilan She, Zhangli Xing, Yuntian Chen, Chunlei He, Pengsheng Wu, Xiangpeng Wang, ZiXuan Kong

**Affiliations:** 1 School of Basic Medical Sciences, Capital Medical University, Beijing, 100069 China; 2 Department of Radiology, the Second Affiliated Hospital of Dalian Medical University, Dalian, 116023 China; 3 Department of Radiology, Peking University First Hospital, Beijing, 100034 China; 4 Department of Radiology, Fujian Medical University Union Hospital, No. 29, Xin Quan Road, Gulou District, Fuzhou, 350001 Fujian Province China; 5 Department of Radiology, West China Hospital, Sichuan University, Chengdu, 610041 China; 6 Beijing Smart Tree Medical Technology Co. Ltd., No. 97, Changping Road, Shahe Town, Changping District Beijing, 102200 China

**Keywords:** Gleason score, Prostate cancer, Multiparametric magnetic resonance imaging, Deep learning, Radiomics

## Abstract

**Objective:**

To evaluate the feasibility of utilizing artificial intelligence (AI)-predicted biparametric MRI (bpMRI) image features for predicting the aggressiveness of prostate cancer (PCa).

**Materials and methods:**

A total of 878 PCa patients from 4 hospitals were retrospectively collected, all of whom had pathological results after radical prostatectomy (RP). A pre-trained AI algorithm was used to select suspected PCa lesions and extract lesion features for model development. The study evaluated five prediction methods, including (1) A clinical-imaging model of clinical features and image features of suspected PCa lesions selected by AI algorithm, (2) the PIRADS category, (3) a conventional radiomics model, (4) a deep-learning bases radiomics model, and (5) biopsy pathology.

**Results:**

In the externally validated dataset, the deep learning-based radiomics model showed the highest area under the curve (AUC 0.700 to 0.791). It exceeded the clinical-imaging model (AUC 0.597 to 0.718), conventional radiomic model (AUC 0.566 to 0.632), PIRADS score (AUC 0.554 to 0.613), and biopsy pathology (AUC 0.537 to 0.578). The AUC predicted by the model did not show a statistically significant difference among the three externally verified hospitals (*p* > 0.05).

**Conclusion:**

Deep-learning radiomics models utilizing AI-extracted image features from bpMRI images can potentially be used to predict PCa aggressiveness, demonstrating a generalized ability for external validation.

**Critical relevance statement:**

Predicting the aggressiveness of prostate cancer (PCa) is important for formulating the best treatment plan for patients. The radiomic model based on deep learning is expected to provide an objective and non-invasive method for evaluating the aggressiveness of PCa.

**Key Points:**

- Predicting the aggressiveness of PCa is important for patients to obtain the best treatment options.
- The deep learning-based radiomics model can predict the aggressiveness of PCa with high accuracy.
- The model has good universality when tested on multiple external datasets.

**Graphical Abstract:**

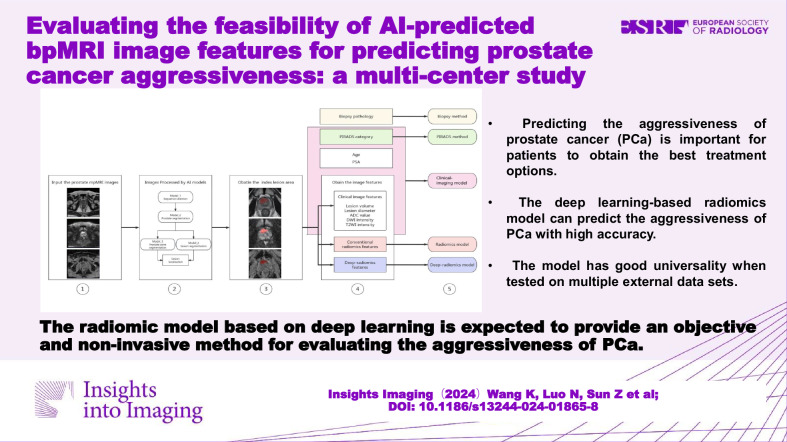

## Introduction

Prostate cancer (PCa) is the second most common cancer in men worldwide [1]. At present, avoiding overtreatment of inert PCa is a clinical challenge [2]. Since the selection of a PCa treatment plan and its prognostic value are closely related to its biological behavior, early prediction and accurate judgment of whether the patient’s lesion is invasive are clinically important for obtaining the best treatment plan and effect [3].

Gleason score (GS) is a commonly used and effective indicator of the invasiveness of PCa. In 2014, the International Society of Urology Pathology (ISUP) proposed the use of the Gleason grade group (GG), which was divided into five groups according to the GS, which can better guide clinical work [4]. Transrectal ultrasound (TRUS)-guided biopsy is often used to determine preoperative GS, but due to the randomness of biopsy sampling, it was not possible to accurately evaluate PCa lesions and evaluate tumor heterogeneity. For the above reasons, GS obtained by preoperative biopsy is different from GS obtained by radical prostatectomy (RP), which may cause patients to be over- or undertreated [5, 6]. Advances in prostate multiparametric magnetic resonance imaging (mpMRI) and prostate imaging reporting and data system (PI-RADS v2.1) have improved the ability to detect and evaluate PCa. Considering the limited utility of dynamic contrast-enhanced (DCE) MRI in assigning PI-RADS scores, some experts have proposed eliminating DCE from the protocol, leading to the concept of biparametric MRI (bpMRI), which relies solely on T2-weighted imaging (T2WI) and diffusion-weighted imaging (DWI). The advantages of bpMRI are believed to include reduced costs and shorter examination durations due to the exclusion of the DCE sequence. Related research [7] has shown that the apparent diffusion coefficient (ADC) of the PCa region is a better indicator of PCa aggressiveness than the TRUS biopsy GS. However, the analysis of prostate bpMRIs relies mainly on the experience and subjective judgment of radiologists, resulting in increased variability and decreased reliability of the results [8].

Radiomics research involves the extraction of high-throughput and quantitative features from multimodal medical images and the use of machine-learning algorithms to transform these features into high-dimensional mining information related to tumor pathophysiology, which may aid clinical diagnosis and decision-making [9, 10]. Radiomic models based on mpMRI have been used for risk stratification of PCa patients [11–14]. Several studies have shown that the features extracted from T2WI and ADC are helpful for classifying the GS [15–17]. At present, manual delineation of the region of interest (ROI) is still the main method of radiometric analysis and inevitably consumes certain human resources and time. Most related research reports [13, 18, 19] at this stage are limited to single-center research. The generalization ability of relevant models and how to reach the level of clinical application still need to be discussed and verified.

In this study, we used a pre-trained artificial intelligence (AI) algorithm to automatically segment PCa lesions [20] and extract bpMRI features. We developed various models and evaluated their feasibility for predicting PCa invasiveness before surgery. In addition, the clinical efficacy of the prediction model was evaluated and validated on a multi-center dataset.

## Materials and methods

### Data enrollment

**Fig. 1:**
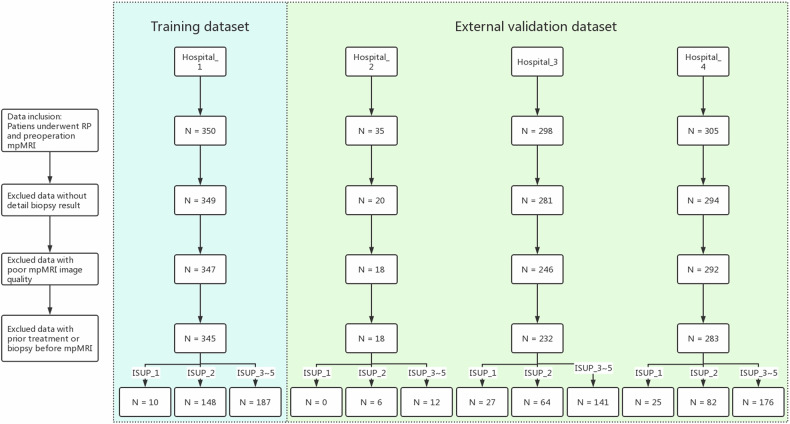
Data enrollment process. Flowchart depicting the process of data inclusion. The training dataset comprises data from Hospital_1, while the external validation dataset includes data from Hospital_2, Hospital_3, and Hospital_4. All the data adhered to the same inclusion and exclusion criteria. In total, the training dataset consisted of 345 included patients, and the external validation dataset included 533 patients: 18 from Hospital_2, 231 from Hospital_3, and 284 from Hospital_4

This retrospective study received approval from the institutional review board (IRB number: 2021060), and all methods were performed in accordance with the relevant guidelines. The statement granted a waiver for written patient informed consent. The data were collected retrospectively from four distinct hospitals. The study included patients who underwent prostate bpMRI followed by RP between November 2017 and December 2022. This encompassed gathering bpMRI images and pertinent clinical information, such as age, PSA levels, bpMRI results, biopsy pathology, and RP pathology reports. The exclusion criteria were as follows: (1) prior endocrine therapy, (2) benign prostate hyperplasia according to RP pathology, (3) missing PSA data, (4) incomplete biopsy pathology records, (5) incomplete MR images, and (6) evident image artifacts. The process of data enrollment is depicted in Fig. 1.

### Reference standard

All patients underwent both biopsy and RP, and pathology samples were available for all patients. Pathology was assessed by experienced pathologists following the guidelines of the International Society of Urological Pathology (ISUP) group. The reference standard was established using the RP pathology results, categorizing the patients into three groups: ISUP_1, ISUP_2, and ISUP_3–5.

### MR scanning protocols

The bpMRI images in this study were acquired with 13 MR scanners from five different vendors. Detailed information regarding the MR scanners and image acquisition protocols can be found in Table 1.

**Table 1:** MR scanning protocols used in different hospitals

	Overall	Training dataset	External validation dataset (*N* = 533)			*p-*value
	(*N* = 878)	Hospital_1	Hospital_2	Hospital_3	Hospital_4	
		(*N* = 345)	(*N* = 18)	(*N* = 231)	(*N* = 284)	
MR scanner						
Magnetic field						< 0.001
1.436 T	5 (0.6%)	0 (0%)	0 (0%)	0 (0%)	5 (1.8%)	
1.5 T	229 (26.1%)	19 (5.5%)	0 (0%)	45 (19.5%)	165 (58.1%)	
3.0 T	644 (73.3%)	326 (94.5%)	18 (100%)	186 (80.5%)	114 (40.1%)	
Station name						< 0.001
GE-Scanner_1	262 (29.8%)	262 (75.9%)	0 (0%)	0 (0%)	0 (0%)	
GE-Scanner_2	37 (4.2%)	0 (0%)	0 (0%)	37 (16.0%)	0 (0%)	
GE-Scanner_3	159 (18.1%)	0 (0%)	1 (5.6%)	44 (19.0%)	114 (40.1%)	
PHILIPS_Scanner_1	38 (4.3%)	38 (11.0%)	0 (0%)	0 (0%)	0 (0%)	
PHILIPS_Scanner_2	1 (0.1%)	1 (0.3%)	0 (0%)	0 (0%)	0 (0%)	
PHILIPS_Scanner_3	1 (0.1%)	1 (0.3%)	0 (0%)	0 (0%)	0 (0%)	
SIEMENS_Scanner_1	14 (1.6%)	0 (0%)	14 (77.8%)	0 (0%)	0 (0%)	
SIEMENS_Scanner_2	43 (4.9%)	0 (0%)	0 (0%)	43 (18.6%)	0 (0%)	
SIEMENS_Scanner_3	64 (7.3%)	0 (0%)	0 (0%)	64 (27.7%)	0 (0%)	
SIEMENS_Scanner_4	19 (2.2%)	19 (5.5%)	0 (0%)	0 (0%)	0 (0%)	
SIEMENS_Scanner_5	3 (0.3%)	0 (0%)	3 (16.7%)	0 (0%)	0 (0%)	
SIEMENS_Scanner_6	208 (23.7%)	0 (0%)	0 (0%)	43 (18.6%)	165 (58.1%)	
UIH_Scanner_1	29 (3.3%)	24 (7.0%)	0 (0%)	0 (0%)	5 (1.8%)	
DWI/ADC protocol						
*B*-value (10^-6^ s/mm^2^)						< 0.001
800	8 (0.9%)	6 (1.7%)	1 (5.6%)	0 (0%)	1 (0.4%)	
1000	288 (32.8%)	0 (0%)	2 (11.1%)	8 (3.5%)	278 (97.9%)	
1200	97 (11.0%)	0 (0%)	0 (0%)	96 (41.6%)	1 (0.4%)	
1400	377 (42.9%)	285 (82.6%)	1 (5.6%)	87 (37.7%)	4 (1.4%)	
1500	52 (5.9%)	0 (0%)	12 (66.7%)	40 (17.3%)	0 (0%)	
2000	56 (6.4%)	54 (15.7%)	2 (11.1%)	0 (0%)	0 (0%)	
Repetition time (ms)						< 0.001
Median (Q1, Q3)	3000 (2630, 3800)	2680 (2650, 3000)	2500 (2500, 3660)	4310 (3000, 4850)	3000 (2090, 3000)	
Echo time (ms)						< 0.001
Median (Q1, Q3)	61.7 (59.9, 70.0)	61.0 (59.8, 61.5)	91.0 (82.8, 91.0)	70.0 (63.0, 74.0)	70.0 (59.7, 70.0)	
Pixel bandwidth (MHz)						< 0.001
Median (Q1, Q3)	1950 (1540, 1950)	1950 (1950, 1950)	1300 (1300, 1500)	1630 (1540, 1950)	1540 (1540, 1950)	
Slice thickness (mm)						< 0.001
Median (Q1, Q3)	3.50 (3.00, 4.00)	4.00 (4.00, 4.00)	4.00 (4.00, 4.00)	3.00 (3.00, 3.50)	3.50 (3.00, 3.50)	
Slice spacing (mm)						< 0.001
Median (Q1, Q3)	4.00 (3.50, 4.00)	4.00 (4.00, 4.00)	4.80 (4.80, 4.80)	3.60 (3.30, 4.00)	3.50 (3.50, 4.00)	
Reconstruction diameter (ms)						< 0.001
Median (Q1, Q3)	230 (200, 240)	240 (220, 240)	250 (250, 250)	221 (200, 260)	200 (200, 340)	
Pixel spacing (ms)						< 0.001
Median (Q1, Q3)	1.25 (0.938, 1.63)	0.938 (0.938, 0.938)	1.30 (1.30, 1.30)	1.33 (0.877, 1.63)	1.56 (1.33, 1.79)	
T2WI protocol						
Repetition time (ms)						< 0.001
Median (Q1, Q3)	3560 (3130, 4470)	3130 (3020, 3500)	4000 (4000, 4000)	3710 (3340, 4120)	5400 (3410, 5540)	
Echo time (ms)						< 0.001
Median (Q1, Q3)	105 (88.9, 112)	88.8 (87.3, 93.0)	100 (100, 100)	108 (96.0, 112)	112 (106, 112)	
Pixel bandwidth (MHz)						< 0.001
Median (Q1, Q3)	163 (163, 200)	163 (163, 163)	203 (203, 203)	190 (160, 200)	200 (163, 200)	
Slice thickness (mm)						< 0.001
Median (Q1, Q3)	3.50 (3.00, 4.00)	4.00 (4.00, 4.00)	3.00 (3.00, 3.00)	3.00 (3.00, 3.50)	3.50 (3.00, 3.50)	
Slice spacing (mm)						< 0.001
Median (Q1, Q3)	4.00 (3.50, 4.00)	4.00 (4.00, 4.00)	3.60 (3.60, 3.60)	3.60 (3.50, 4.00)	3.50 (3.50, 4.00)	
Reconstruction diameter (ms)						< 0.001
Median (Q1, Q3)	200 (200, 240)	240 (240, 240)	178 (178, 178)	200 (200, 220)	200 (200, 200)	
Pixel spacing (ms)						< 0.001
Median (Q1, Q3)	0.469 (0.391, 0.625)	0.469 (0.469, 0.469)	0.625 (0.625, 0.625)	0.391 (0.344, 0.625)	0.625 (0.391, 0.625)	

### Image features extracted by AI algorithms

Following anonymization, the DICOM files were converted to NIFTI format using dicom2nii.py (Python 3.5) and then input into pre-trained AI software. For detailed technical information about the AI software, please refer to the supplementary material.

**Fig. 2:**
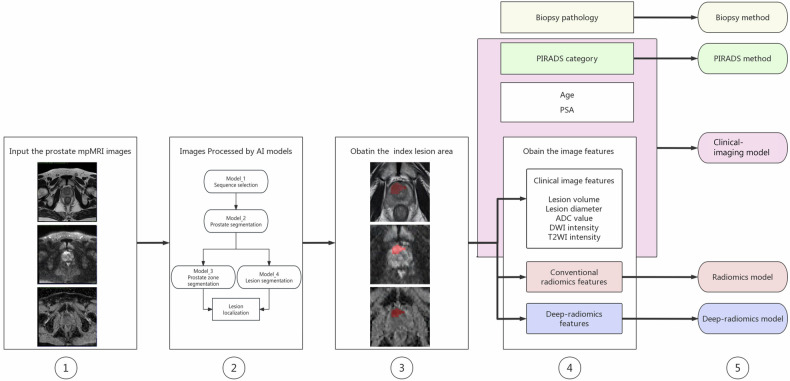
Extraction of image features and construction of prediction methods. Step 1 involves selecting and inputting anonymized mpMRI images, which encompass T2WI, DWI, and ADC maps. In Step 2, the pre-trained AI models are employed to identify suspicious areas indicative of PCa. For a detailed process, see the supplementary materials. Step 3 entails identifying the index lesion by selecting the largest lesion from the predicted labels. The index lesion was annotated as the red areas on the images. Step 4 encompasses extracting image features from the index lesion, encompassing (1) clinical image features such as lesion volume, lesion diameter, ADC value, DWI signal intensity, and T2WI intensity, (2) conventional radiomic features, and (3) deep-radiomic features. In Step 5, various prediction methods are developed, including (a) biopsy prediction, which forecasts ISUP grouping based on biopsy pathology; (b) PIRADS prediction, which anticipates ISUP grouping based on the PIRADS category; (c) a clinical-imaging model, which incorporates age, PSA, PIRADS category, and clinical image features to predict ISUP grouping; (d) radiomics model, which forecasts ISUP grouping using conventional radiomic features obtained from the index lesion; and (e) deep-radiomics model, which forecasts ISUP grouping using deep-radiomic features extracted from the index lesion

For each enrolled patient, the largest lesion predicted by the AI algorithms on the bpMRI images was designated the index lesion for analysis. The lesion’s location was classified as solely in the peripheral zone (PZ), solely in the transitional zone (TZ), or in both the PZ and TZ (PZ + TZ). The following image features were computed and utilized in the prediction model: (1) lesion volume, (2) diameter of the lesion, signifying the largest diameter of the lesion, (3) mean ADC value of the lesion (ADClesion), (4) mean signal intensity of the lesion on DWI (DWIlesion), and 5) mean signal intensity of the lesion on T2WI (T2WIlesion) (as illustrated in Fig. 2).

### Prediction methods

To predict the ISUP_1, ISUP_2, and ISUP_3–5 classes, five prediction methods were developed, namely, the PIRADS category, biopsy pathology, a clinical-imaging model, a radiomics model, and a deep-radiomics model. The steps involved in developing the prediction methods are illustrated in Fig. 2. The training dataset utilized was sourced from Hospital_1, while the external validation dataset included data from Hospital_2, Hospital_3, and Hospital_4.

Both the PIRADS and biopsy methods use their results to fit an ordinal logistic regression model to predict the final PR pathology grading. Specifically, the PIRADS method uses the PIRADS categories (1 to 5) to predict the probability of the final PR pathology being classified as ISUP_1, ISUP_2, or ISUP_3–5. The biopsy method uses biopsy results (negative or ISUP 1 to 5) to predict the probability of the final PR pathology being classified into the same ISUP categories.

For the construction of the clinical-imaging model, clinical variables were employed as covariates. These included age, PSA levels, PIRADS score, and image features of the suspected clinically significant prostate cancer (csPCa) lesion, which included position, volume, diameter, ADC value, signal intensity on DWI, and signal intensity on T2WI.

To construct the radiomics model, image features were derived from regions of interest (ROIs) on the ADC maps using the PyRadiomics package in Python (https://pyradiomics.readthedocs.io/en/latest/changes.html). *Z*-score normalization was applied to standardize the extracted features, and Pearson correlation coefficients (PCCs) were computed to identify highly correlated features. Features with a PCC value exceeding 0.99 were eliminated to mitigate multicollinearity. The Kruskal‒Wallis test was then employed to select the features for the final radiomics model. For the classifier, the least absolute shrinkage and selection operator (LASSO) algorithm was utilized.

To construct the deep-radiomics model, image features were extracted using a deep-learning algorithm. The construction process involved several key steps: (1) Preprocessing ADC maps: Initial preprocessing included normalizing the intensities of the ADC maps. (2) ROI resampling: ROIs were resampled to ensure a consistent voxel size. (3) Deep feature extraction: A pre-trained deep-learning model was used to extract features from the segmented ROIs. (4) Feature dimension reduction: The resulting channel feature maps underwent dimension reduction by filtering with the maximum value, resulting in a set of 512 one-dimensional features. (5) Model construction: After extracting the deep features, the construction of the deep-radiomics model followed a procedure similar to that of the radiomics model, as previously described.

For model tuning, 5-fold cross-validation was employed to select the optimal value of the hyperparameter *α*, which controls the strength of the L1 penalty. A grid search was performed over a range of *α* values, and the one yielding the highest cross-validated accuracy was chosen. This process identified a subset of significant predictors associated with ISUP classes.

### Prediction efficacy evaluation

The external dataset was used to assess the prediction effectiveness of the methods via receiver operating characteristic (ROC) analysis. This evaluation was conducted by computing the area under the ROC curve (AUC).

### Statistical analysis

The statistical analysis was conducted using R 4.1.3 software. Quantitative variables with a normal distribution are presented as the mean ± standard deviation, while those with a nonnormal distribution are presented as the median (Q1, Q3). Categorical variables are presented as frequencies. The normality of the variables was assessed using the Kolmogorov‒Smirnov test, associations between categorical variables were examined using the chi-square test, and differences between multiple groups were compared using the Kruskal‒Wallis test. Multiclass ROC analysis was performed using the multiclass.roc() function from the pROC package. The calculation of classification metrics was performed using the reportROC() function from the reportROC package (supplementary material). The DeLong test was used to compare differences in the area under the curve (AUC) among the five types of prediction methods. A *p*-value less than 0.05 was considered to indicate statistical significance.

## Results

### Clinical characteristics

A total of 878 eligible patients participated in the study, with an average age of 68.7 ± 6.6 years. Among these patients, 345 were recruited from Hospital_1, and 18, 231, and 284 were from Hospital_2, Hospital_3, and Hospital_4, respectively. All clinical variables exhibited statistically significant differences among the four hospitals (all *p* < 0.001), except for age (*p* = 0.878). Further details regarding the clinical variables among the different datasets can be found in Table 2.

**Table 2:** Clinical characteristics of patients in the training and external validation cohorts

	Overall	Training cohort	External validation cohort			*p-*value
	(*N* = 878)	Hospital_1	Hospital_2	Hospital_3	Hospital_4	
		(*N* = 345)	(*N* = 18)	(*N* = 231)	(*N* = 284)	
Age (year)						0.878
Mean (SD)	68.7 (6.63)	68.5 (6.58)	69.4 (6.71)	68.8 (6.73)	68.7 (6.64)	
PSA (ng/dL)						< 0.001
Median (Q1, Q3)	11.6 (7.90, 19.1)	10.9 (7.06, 15.5)	9.93 (6.35, 14.0)	12.8 (8.82, 23.9)	12.8 (8.20, 22.5)	
PIRADS						
PIRADS_1	2 (0.2%)	1 (0.3%)	0 (0%)	1 (0.4%)	0 (0%)	< 0.001
PIRADS_2	87 (9.9%)	12 (3.5%)	4 (22.2%)	4 (1.7%)	67 (23.6%)	
PIRADS_3	216 (24.6%)	45 (13.0%)	8 (44.4%)	57 (24.7%)	106 (37.3%)	
PIRADS_4	286 (32.6%)	173 (50.1%)	0 (0%)	57 (24.7%)	56 (19.7%)	
PIRADS_5	287 (32.7%)	114 (33.0%)	6 (33.3%)	112 (48.5%)	55 (19.4%)	
PCa location						< 0.001
Not measurable	137 (15.6%)	110 (31.9%)	1 (5.6%)	12 (5.2%)	14 (4.9%)	
PZ	80 (9.1%)	38 (11.0%)	3 (16.7%)	14 (6.1%)	25 (8.8%)	
PZ + TZ	491 (55.9%)	121 (35.1%)	10 (55.6%)	172 (74.5%)	188 (66.2%)	
TZ	170 (19.4%)	76 (22.0%)	4 (22.2%)	33 (14.3%)	57 (20.1%)	
Lesion volume						< 0.001
Median (Q1, Q3)	1.60 (0.717, 3.93)	0.737 (0, 1.55)	2.73 (0.976, 4.68)	3.25 (1.44, 7.62)	2.44 (1.16, 5.25)	
Lesion diameter						< 0.001
Median (Q1, Q3)	24.9 (17.8, 33.5)	18.2 (0, 25.4)	27.5 (23.7, 37.2)	31.1 (23.1, 40.0)	28.1 (21.3, 36.2)	
ADC_lesion_ (10^6 ^ mm^2^/s)						< 0.001
Median (Q1, Q3)	802 (663, 907)	738 (0, 865)	848 (757, 957)	816 (714, 909)	840 (739, 926)	
DWI_lesion_						< 0.001
Median (Q1, Q3)	120 (46.3, 404)	52.2 (0, 85.7)	313 (176, 398)	266 (80.5, 510)	281 (102, 506)	
TWI_lesion_						< 0.001
Median (Q1, Q3)	324 (119, 1150)	127 (0, 297)	579 (436, 1490)	490 (160, 1580)	499 (274, 1520)	
Biopsy result						< 0.001
No cancer	8 (0.9%)	0 (0%)	0 (0%)	1 (0.4%)	7 (2.5%)	
ISUP_1	132 (15.0%)	49 (14.2%)	0 (0%)	14 (6.1%)	69 (24.3%)	
ISUP_2	185 (21.1%)	128 (37.1%)	0 (0%)	18 (7.8%)	39 (13.7%)	
ISUP_3	331 (37.7%)	84 (24.3%)	18 (100%)	159 (68.8%)	70 (24.6%)	
ISUP_4	133 (15.1%)	43 (12.5%)	0 (0%)	26 (11.3%)	64 (22.5%)	
ISUP_5	89 (10.1%)	41 (11.9%)	0 (0%)	13 (5.6%)	35 (12.3%)	
PR result						
ISUP_1	62 (7.1%)	10 (2.9%)	0 (0%)	27 (11.7%)	25 (8.8%)	< 0.001
ISUP_2	300 (34.2%)	148 (42.9%)	6 (33.3%)	63 (27.3%)	83 (29.2%)	
ISUP_3	241 (27.4%)	93 (27.0%)	3 (16.7%)	66 (28.6%)	79 (27.8%)	
ISUP_4	108 (12.3%)	40 (11.6%)	4 (22.2%)	27 (11.7%)	37 (13.0%)	
ISUP_5	167 (19.0%)	54 (15.7%)	5 (27.8%)	48 (20.8%)	60 (21.1%)	

**Fig. 3:**
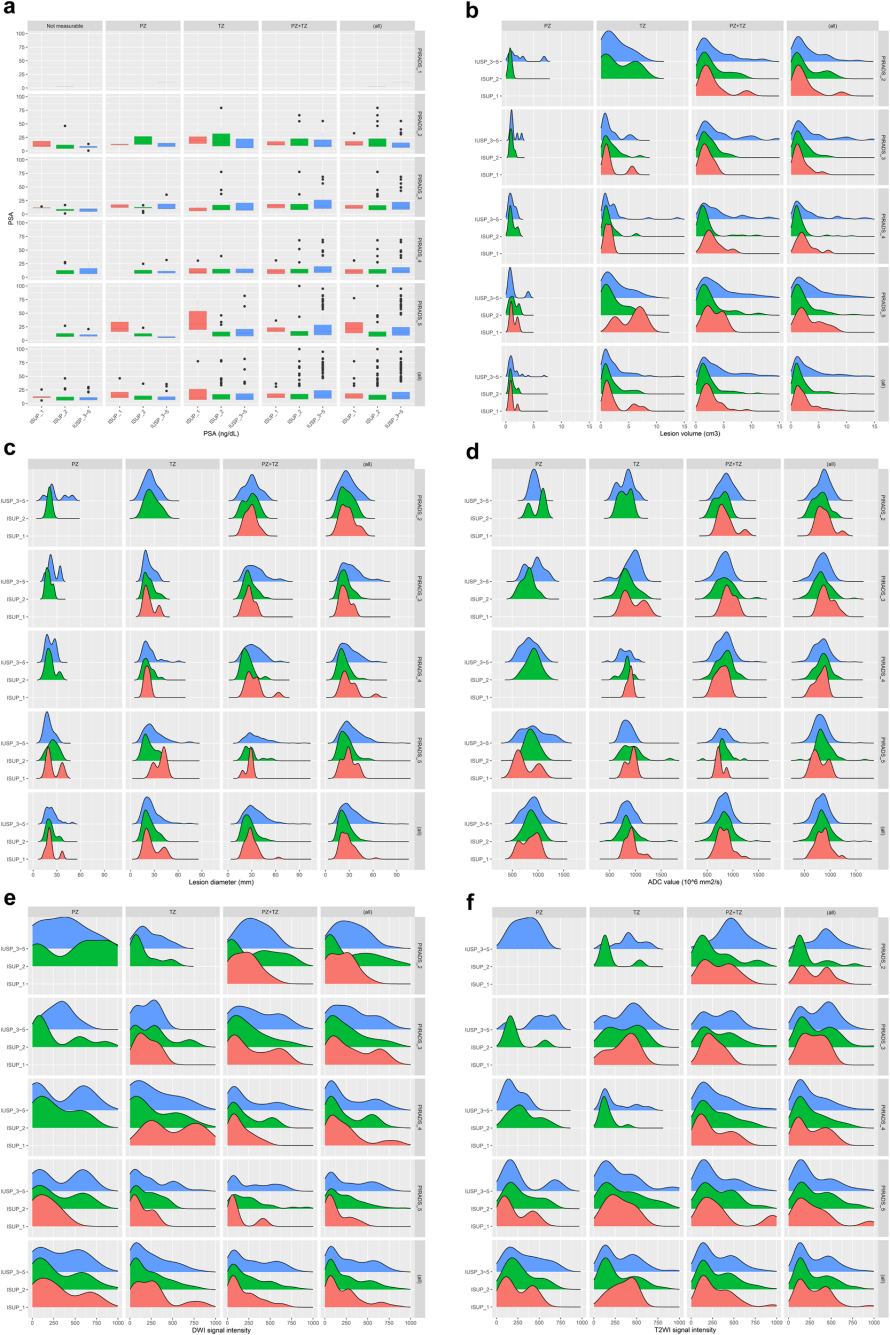
Distribution of the clinical variables among the different ISUP classes. **a** displays the distribution of PSA levels. Columns 1 to 4 show the PSA values for lesions classified as “not measurable,” lesions in the PZ, lesions in the TZ, and lesions in both the PZ and TZ, respectively. Column 5 displays the PSA values for all lesions. Rows 1 to 5 display the PSA values for lesions categorized as PIRADS_1 to PIRADS_5, and row 6 shows the PSA values for all lesions. **b** to **f** demonstrate the distribution of measurements for lesion volume, lesion diameter, ADC value, DWI signal intensity, and T2WI signal intensity, with the similar manner. Columns 1 to 3 display the measurements for lesions in the PZ, lesions in the TZ, and lesions in both the PZ and TZ, respectively. Column 4 represents the measurements for all lesions. When observed by rows, rows 1 to 4 show the measurements for lesions categorized as PIRADS_2 to PIRADS_5, and row 5 displays the measurements for all lesions

Based on the RP pathology results, 62 patients were categorized as ISUP_1, 300 as ISUP_2, and 516 as ISUP_3–5. Except for patient age distribution, which did not differ significantly among the three ISUP classes (*p *= 0.563), all the other clinical variables demonstrated significant differences among the various ISUP classes (all *p* < 0.05). Moreover, there was a statistically significant difference in the distribution of ISUP classes among the four hospitals (*p* < 0.001). Detailed information about the variables in different ISUP classes can be found in Table 3. The distributions of these variables across different ISUP classes are illustrated in Fig. 3.

**Table 3:** Clinical characteristics of patients in different ISUP classes

	Overall	ISUP_1	ISUP_2	ISUP_3–5	*p*-value
	(*N* = 878)	(*N* = 62)	(*N* = 300)	(*N* = 516)	
Age (year)					0.563
Mean (SD)	68.7 (6.63)	69.6 (6.91)	68.5 (6.74)	68.6 (6.54)	
PSA (ng/dL)					< 0.001
Median (Q1, Q3)	11.6 (7.90, 19.1)	11.9 (9.37, 18.4)	10.8 (6.99, 15.8)	12.3 (8.18, 21.4)	
PIRADS					< 0.001
PIRADS_1	2 (0.2%)	0 (0%)	1 (0.3%)	1 (0.2%)	
PIRADS_2	87 (9.9%)	16 (25.8%)	29 (9.7%)	42 (8.1%)	
PIRADS_3	216 (24.6%)	20 (32.3%)	81 (27.0%)	115 (22.3%)	
PIRADS_4	286 (32.6%)	15 (24.2%)	119 (39.7%)	152 (29.5%)	
PIRADS_5	287 (32.7%)	11 (17.7%)	70 (23.3%)	206 (39.9%)	
PCa location					< 0.001
Not measurable	137 (15.6%)	7 (11.3%)	86 (28.7%)	44 (8.5%)	
PZ	80 (9.1%)	7 (11.3%)	39 (13.0%)	34 (6.6%)	
TZ	170 (19.4%)	14 (22.6%)	67 (22.3%)	89 (17.2%)	
PZ + TZ	491 (55.9%)	34 (54.8%)	108 (36.0%)	349 (67.6%)	
Lesion volume					< 0.001
Median (Q1, Q3)	1.60 (0.717, 3.93)	1.66 (0.869, 2.84)	0.887 (0, 1.90)	2.37 (0.973, 5.87)	
Lesion diameter					< 0.001
Median (Q1, Q3)	24.9 (17.8, 33.5)	25.4 (19.1, 31.1)	19.6 (0, 26.1)	28.1 (20.6, 38.1)	
ADC_lesion_ (10^6^ mm^2^/s)					< 0.001
Median (Q1, Q3)	802 (663, 907)	820 (732, 915)	773 (0, 892)	813 (697, 907)	
DWI_lesion_					< 0.001
Median (Q1, Q3)	120 (46.3, 404)	101 (54.5, 282)	68.6 (0, 287)	205 (61.4, 489)	
TWI_lesion_					< 0.001
Median (Q1, Q3)	324 (119, 1150)	303 (122, 476)	162 (0, 576)	434 (132, 1390)	
Biopsy result					< 0.001
No cancer	8 (0.9%)	2 (3.2%)	1 (0.3%)	5 (1.0%)	
ISUP_1	132 (15.0%)	33 (53.2%)	62 (20.7%)	37 (7.2%)	
ISUP_2	185 (21.1%)	3 (4.8%)	111 (37.0%)	71 (13.8%)	
ISUP_3	331 (37.7%)	22 (35.5%)	99 (33.0%)	210 (40.7%)	
ISUP_4	133 (15.1%)	2 (3.2%)	21 (7.0%)	110 (21.3%)	
ISUP_5	89 (10.1%)	0 (0%)	6 (2.0%)	83 (16.1%)	
Dataset					< 0.001
Training dataset	345 (39.3%)	10 (16.1%)	148 (49.3%)	187 (36.2%)	
Test dataset	533 (60.7%)	52 (83.9%)	152 (50.7%)	329 (63.8%)	
Institute name					< 0.001
Hospital_1	345 (39.3%)	10 (16.1%)	148 (49.3%)	187 (36.2%)	
Hospital_2	18 (2.1%)	0 (0%)	6 (2.0%)	12 (2.3%)	
Hospital_3	231 (26.3%)	27 (43.5%)	63 (21.0%)	141 (27.3%)	
Hospital_4	284 (32.3%)	25 (40.3%)	83 (27.7%)	176 (34.1%)

### Model development metrics

Ordinal logistic regression analysis results indicated that age, PSA level, lesion location, and the ADC value were statistically significant predictors (all *p* < 0.001), while PIRADS, volume, diameter, DWI intensity, and T2WI intensity did not significantly differ (all *p* > 0.05). The model exhibited a good fit to the data, as evidenced by the Brant test (*p* > 0.05). Additional details of the ordinal logistic regression model can be found in the supplementary material (Table S17).

The radiomics model was constructed using the LASSO method, which identified 13 critical features (3 first-order features and 10 texture features) for inclusion in the final model. The deep-radiomics model incorporated 13 features extracted from the convolutional network. The development of the radiomics and deep-radiomics LASSO classifiers is depicted in the supplementary material (Fig. S8). The coefficients of the selected features for the radiomics model and the deep-radiomics model are presented in the supplementary material (Table S18).

**Fig. 4:**
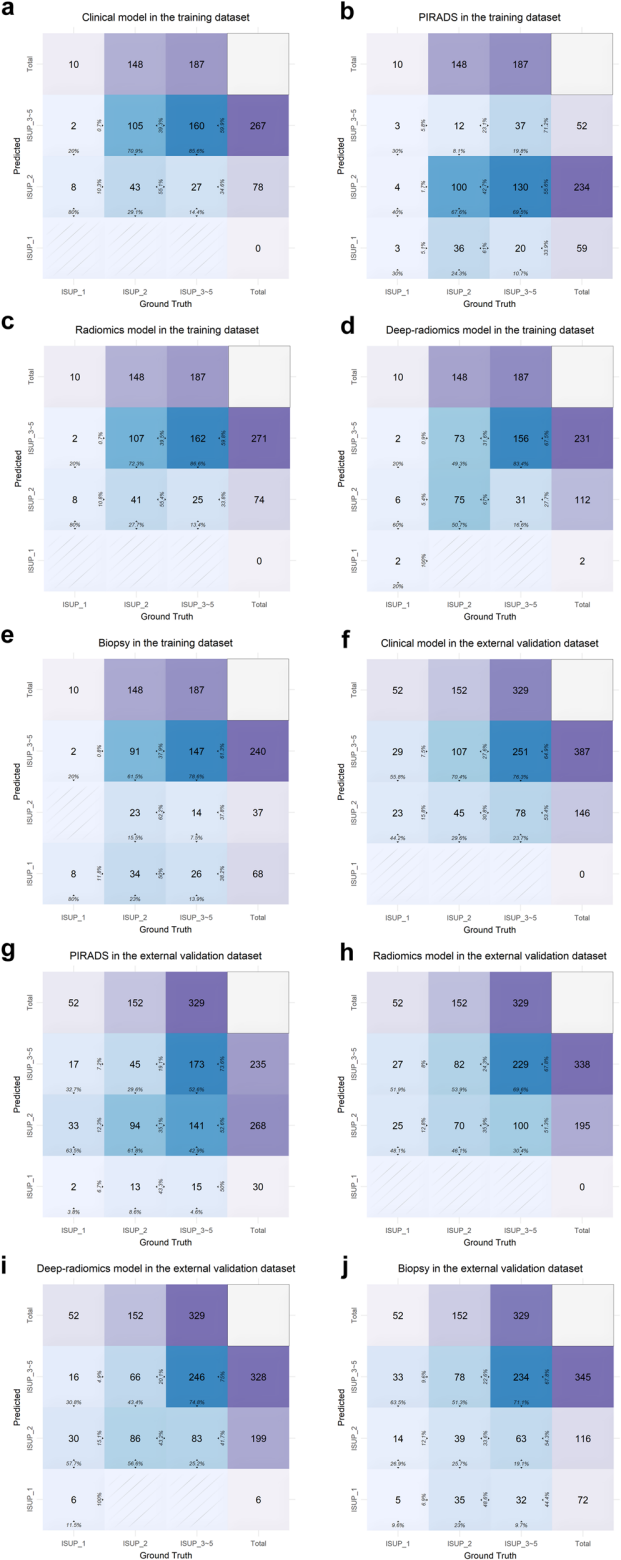
Confusion matrix of the prediction methods. **a** to **e** represents the confusion matrices of the training dataset, while **f** to **j** depicts the confusion matrices of the external validation dataset. **a**, **f** display the predictions of the clinical-imaging model. **b**, **g** show the classification results based on the PIRADS category. **c**, **h** show the predictions of the radiomics model. **d**, **i** show the predictions of the deep-radiomics model. Finally, **e**, **j** illustrate the classification outcomes according to the biopsy results. When observed by columns, the first to three columns display the true values for ISUP_1, ISUP_2, and ISUP_3, respectively, while the fourth column represents the total true values. When observed by rows, from bottom to top, the first to three rows represent the predicted values for ISUP_1, ISUP_2, and ISUP_3, respectively, and the fourth row corresponds to the total predicted values

The prediction results are visualized in the form of a confusion matrix, as illustrated in Fig. 4.

### Model evaluation

**Fig. 5:**
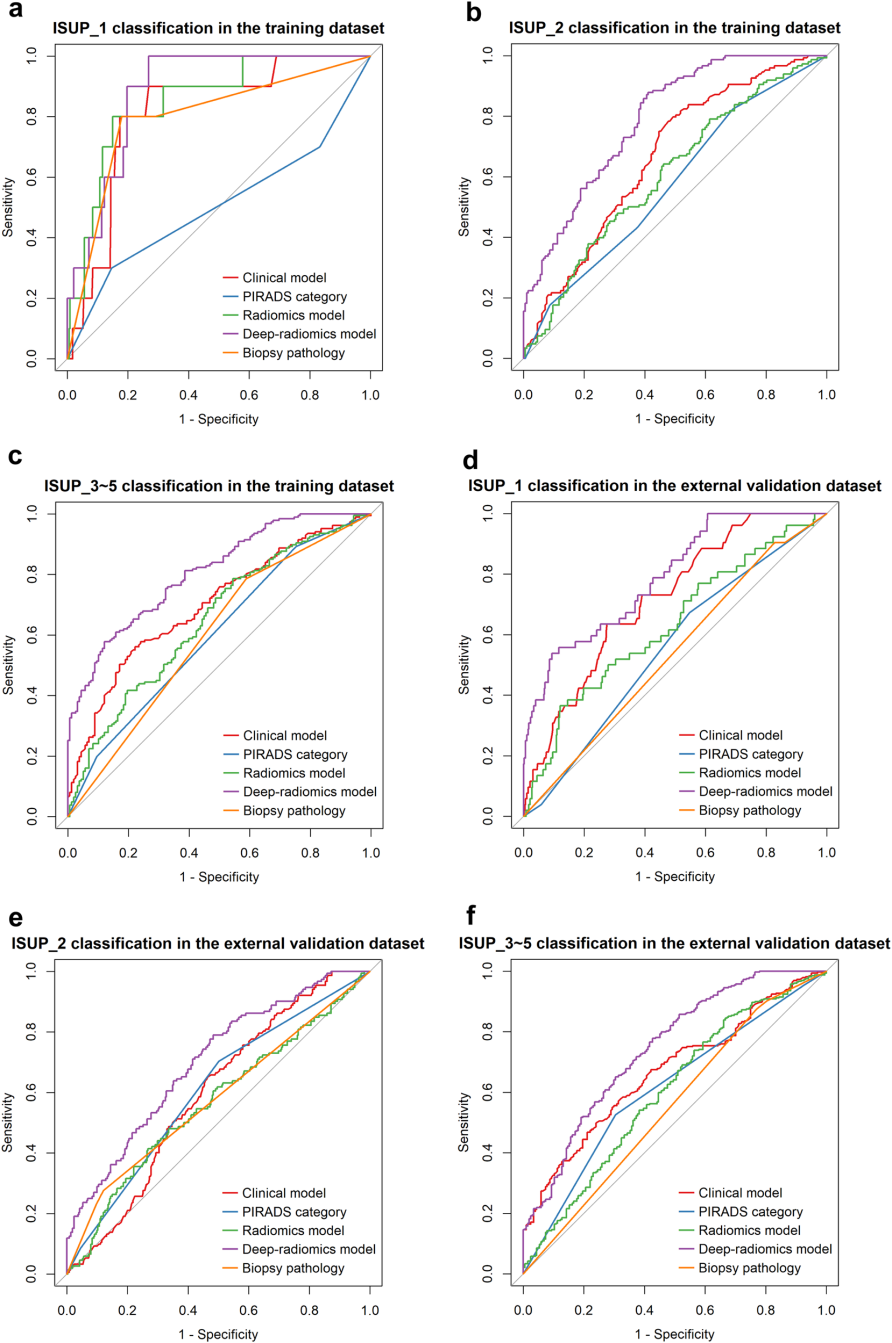
ROC curves of the prediction methods in the training and external validation datasets. **a** to **c** represent the ROC curves of the training dataset, while **d** to **f** depict the ROC curves of the external validation dataset. **a**, **d** display the ROC curves for various prediction methods targeting ISUP_1. **b**, **e** illustrates the ROC curves for various prediction methods targeting ISUP_2. **c**, **f** show the ROC curves for various prediction methods targeting ISUP_3–5

**Fig. 6:**
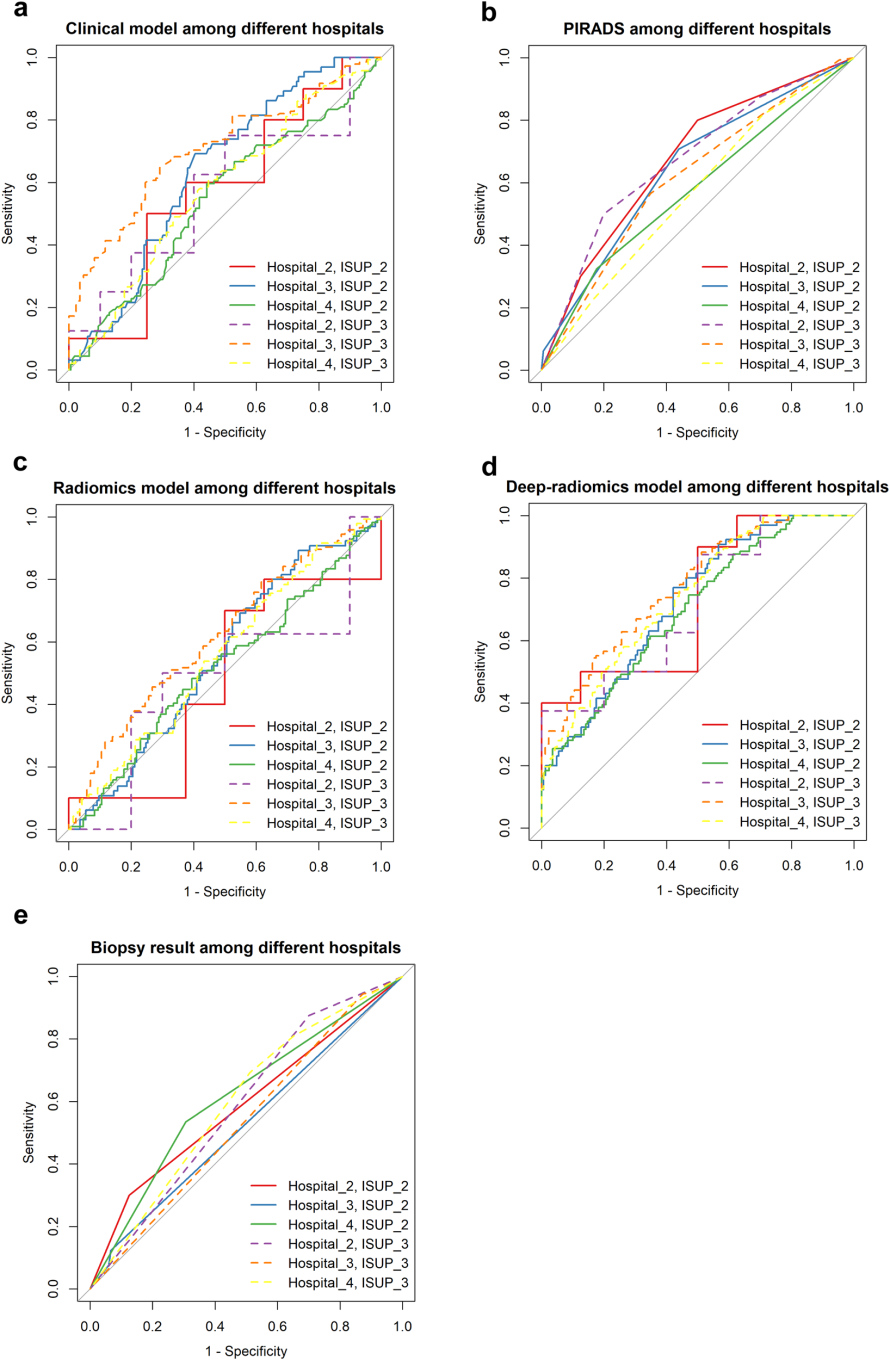
ROC curves of the prediction methods among the hospitals in the external validation cohort. **a** displays the predictions of the clinical-imaging model across different hospitals. **b** shows the classification results based on the PIRADS category across different hospitals. **c** illustrates the predictions of the radiomics model across different hospitals. **d** shows the predictions of the deep-radiomics model across different hospitals. Finally, **e** shows the classification results based on biopsy results across different hospitals

The performance of the clinical-imaging model, PIRADS category, radiomics model, deep-radiomics model, and biopsy pathology was assessed using ROC analysis, and the results are detailed in Figs. 5 and 6 and Tables 4 and 5.

**Table 4:** Efficacy of the five prediction methods in the training and external validation datasets

	AUC	ACC	SEN	SPE	PPV	NPV
Training dataset						
ISUP_1 (*N* = 10)						
Clinical-imaging model	0.815 (0.695, 0.934)	0.736 (0.735, 0.737)	0.900 (0.714, 1.000)	0.731 (0.684, 0.779)	0.091 (0.034, 0.148)	0.996 (0.988, 1.004)
PIRADS category	0.507 (0.293, 0.722)	0.838 (0.837, 0.838)	0.300 (0.016, 0.584)	0.854 (0.816, 0.892)	0.058 (−0.006, 0.121)	0.976 (0.959, 0.994)
Radiomics model	0.852 (0.741, 0.963)	0.849 (0.849, 0.850)	0.800 (0.552, 1.000)	0.851 (0.813, 0.889)	0.138 (0.049, 0.227)	0.993 (0.983, 1.003)
Deep-radiomics model	0.882 (0.819, 0.946)	0.739 (0.738, 0.740)	1.000 (1.000, 1.000)	0.731 (0.684, 0.779)	0.100 (0.041, 0.159)	1.000 (1.000, 1.000)
Biopsy pathology	0.799 (0.653, 0.946)	0.820 (0.819, 0.821)	0.800 (0.552, 1.000)	0.821 (0.780, 0.862)	0.118 (0.041, 0.194)	0.993 (0.983, 1.003)
ISUP_2 (*N* = 148)						
Clinical-imaging model	0.666 (0.609, 0.723)	0.635 (0.633, 0.636)	0.784 (0.717, 0.850)	0.523 (0.453, 0.593)	0.552 (0.485, 0.620)	0.763 (0.691, 0.835)
PIRADS category	0.604 (0.556, 0.653)	0.609 (0.607, 0.610)	0.243 (0.174, 0.312)	0.883 (0.838, 0.928)	0.610 (0.486, 0.735)	0.608 (0.552, 0.665)
Radiomics model	0.609 (0.549, 0.668)	0.583 (0.581, 0.584)	0.642 (0.565, 0.719)	0.538 (0.468, 0.608)	0.511 (0.439, 0.583)	0.667 (0.593, 0.740)
Deep-radiomics model	0.791 (0.745, 0.837)	0.713 (0.712, 0.714)	0.878 (0.826, 0.931)	0.589 (0.520, 0.658)	0.616 (0.550, 0.682)	0.866 (0.808, 0.923)
Biopsy pathology	0.565 (0.515, 0.616)	0.597 (0.596, 0.598)	0.385 (0.307, 0.464)	0.756 (0.696, 0.816)	0.543 (0.448, 0.638)	0.621 (0.559, 0.682)
ISUP_3–5 (*N* = 187)						
Clinical-imaging model	0.699 (0.644, 0.754)	0.661 (0.660, 0.662)	0.561 (0.490, 0.633)	0.778 (0.714, 0.843)	0.750 (0.678, 0.822)	0.600 (0.533, 0.667)
PIRADS category	0.602 (0.553, 0.651)	0.597 (0.596, 0.598)	0.893 (0.849, 0.937)	0.247 (0.180, 0.314)	0.584 (0.527, 0.641)	0.661 (0.540, 0.782)
Radiomics model	0.649 (0.591, 0.707)	0.635 (0.633, 0.636)	0.786 (0.727, 0.845)	0.456 (0.378, 0.533)	0.631 (0.569, 0.693)	0.643 (0.554, 0.732)
Deep-radiomics model	0.808 (0.764, 0.852)	0.716 (0.715, 0.717)	0.578 (0.507, 0.648)	0.880 (0.829, 0.930)	0.850 (0.788, 0.912)	0.638 (0.574, 0.701)
Biopsy pathology	0.599 (0.549, 0.648)	0.614 (0.613, 0.616)	0.786 (0.727, 0.845)	0.411 (0.335, 0.488)	0.613 (0.551, 0.674)	0.619 (0.526, 0.712)
External validation dataset						
ISUP_1 (*N* = 52)						
Clinical-imaging model	0.718 (0.652, 0.785)	0.717 (0.716, 0.717)	0.635 (0.504, 0.765)	0.726 (0.686, 0.765)	0.200 (0.139, 0.261)	0.948 (0.926, 0.971)
PIRADS category	0.554 (0.485, 0.623)	0.475 (0.474, 0.476)	0.673 (0.546, 0.801)	0.453 (0.409, 0.498)	0.117 (0.081, 0.154)	0.928 (0.895, 0.961)
Radiomics model	0.632 (0.549, 0.714)	0.829 (0.829, 0.830)	0.365 (0.235, 0.496)	0.879 (0.850, 0.909)	0.247 (0.150, 0.343)	0.928 (0.904, 0.951)
Deep-radiomics model	0.791 (0.728, 0.854)	0.871 (0.870, 0.871)	0.538 (0.403, 0.674)	0.906 (0.880, 0.932)	0.384 (0.272, 0.495)	0.948 (0.928, 0.968)
Biopsy pathology	0.537 (0.492, 0.582)	0.244 (0.243, 0.245)	0.904 (0.824, 0.984)	0.173 (0.139, 0.206)	0.106 (0.077, 0.134)	0.943 (0.895, 0.992)
ISUP_2 (*N* = 152)						
Clinical-imaging model	0.597 (0.547, 0.646)	0.568 (0.568, 0.569)	0.651 (0.576, 0.727)	0.535 (0.485, 0.586)	0.359 (0.302, 0.415)	0.794 (0.744, 0.843)
PIRADS category	0.607 (0.561, 0.653)	0.557 (0.556, 0.558)	0.704 (0.631, 0.777)	0.499 (0.448, 0.549)	0.359 (0.305, 0.414)	0.809 (0.758, 0.859)
Radiomics model	0.566 (0.511, 0.621)	0.642 (0.641, 0.642)	0.414 (0.336, 0.493)	0.732 (0.688, 0.777)	0.382 (0.308, 0.456)	0.758 (0.714, 0.802)
Deep-radiomics model	0.700 (0.652, 0.748)	0.595 (0.594, 0.596)	0.789 (0.725, 0.854)	0.517 (0.467, 0.567)	0.395 (0.340, 0.450)	0.860 (0.815, 0.905)
Biopsy pathology	0.578 (0.539, 0.618)	0.707 (0.707, 0.708)	0.276 (0.205, 0.347)	0.879 (0.847, 0.912)	0.477 (0.373, 0.582)	0.753 (0.713, 0.793)
ISUP_3–5 (*N* = 329)						
Clinical-imaging model	0.668 (0.622, 0.713)	0.598 (0.598, 0.599)	0.498 (0.444, 0.553)	0.760 (0.701, 0.818)	0.770 (0.713, 0.826)	0.484 (0.430, 0.539)
PIRADS category	0.613 (0.569, 0.656)	0.591 (0.590, 0.592)	0.526 (0.472, 0.580)	0.696 (0.633, 0.759)	0.736 (0.680, 0.793)	0.477 (0.420, 0.533)
Radiomics model	0.602 (0.552, 0.652)	0.649 (0.648, 0.650)	0.845 (0.806, 0.884)	0.333 (0.269, 0.398)	0.671 (0.626, 0.717)	0.571 (0.483, 0.660)
Deep-radiomics model	0.745 (0.703, 0.788)	0.700 (0.699, 0.701)	0.778 (0.733, 0.823)	0.574 (0.506, 0.641)	0.746 (0.700, 0.792)	0.616 (0.547, 0.685)
Biopsy pathology	0.554 (0.520, 0.588)	0.629 (0.628, 0.629)	0.875 (0.840, 0.911)	0.230 (0.173, 0.288)	0.647 (0.603, 0.692)	0.534 (0.430, 0.638)

**Table 5:** Efficacy of the five prediction methods across the different hospitals in the external validation datasets

	AUC	ACC	SEN	SPE	PPV	NPV
Clinical-imaging model						
ISUP_2 (*N* = 152)						
Hospital_2	0.575 (0.285, 0.865)	0.611 (0.585, 0.637)	0.500 (0.190, 0.810)	0.750 (0.450, 1.000)	0.714 (0.380, 1.049)	0.545 (0.251, 0.840)
Hosptial_3	0.638 (0.564, 0.711)	0.623 (0.621, 0.625)	0.692 (0.580, 0.805)	0.596 (0.522, 0.671)	0.402 (0.311, 0.493)	0.832 (0.765, 0.899)
Hosptial_4	0.545 (0.476, 0.615)	0.574 (0.572, 0.576)	0.596 (0.506, 0.687)	0.559 (0.484, 0.633)	0.476 (0.394, 0.557)	0.674 (0.596, 0.751)
ISUP_3–5 (*N* = 329)						
Hospital_2	0.575 (0.285, 0.865)	0.611 (0.585, 0.637)	0.750 (0.450, 1.000)	0.500 (0.190, 0.810)	0.545 (0.251, 0.840)	0.714 (0.380, 1.049)
Hosptial_3	0.706 (0.639, 0.773)	0.671 (0.669, 0.673)	0.648 (0.571, 0.726)	0.709 (0.613, 0.805)	0.790 (0.717, 0.863)	0.545 (0.452, 0.637)
Hosptial_4	0.579 (0.512, 0.645)	0.581 (0.579, 0.583)	0.573 (0.492, 0.654)	0.589 (0.507, 0.670)	0.586 (0.504, 0.667)	0.576 (0.496, 0.657)
PIRADS category						
ISUP_2 (*N* = 152)						
Hospital_2	0.675 (0.429, 0.921)	0.667 (0.642, 0.691)	0.800 (0.552, 1.000)	0.500 (0.154, 0.846)	0.667 (0.400, 0.933)	0.667 (0.289, 1.044)
Hosptial_3	0.616 (0.550, 0.682)	0.597 (0.595, 0.599)	0.566 (0.485, 0.646)	0.651 (0.550, 0.752)	0.732 (0.650, 0.814)	0.471 (0.381, 0.560)
Hosptial_4	0.577 (0.515, 0.639)	0.623 (0.622, 0.625)	0.325 (0.239, 0.411)	0.824 (0.766, 0.881)	0.552 (0.433, 0.671)	0.645 (0.581, 0.709)
ISUP_3–5 (*N* = 329)						
Hospital_2	0.675 (0.429, 0.921)	0.667 (0.642, 0.691)	0.500 (0.154, 0.846)	0.800 (0.552, 1.000)	0.667 (0.289, 1.044)	0.667 (0.400, 0.933)
Hosptial_3	0.616 (0.550, 0.682)	0.597 (0.595, 0.599)	0.566 (0.485, 0.646)	0.651 (0.550, 0.752)	0.732 (0.650, 0.814)	0.471 (0.381, 0.560)
Hosptial_4	0.567 (0.508, 0.626)	0.556 (0.555, 0.558)	0.818 (0.755, 0.881)	0.291 (0.216, 0.366)	0.539 (0.473, 0.605)	0.612 (0.495, 0.729)
Radiomics model						
ISUP_2 (*N* = 152)						
Hospital_2	0.525 (0.227, 0.823)	0.667 (0.642, 0.691)	0.900 (0.714, 1.000)	0.375 (0.040, 0.710)	0.643 (0.392, 0.894)	0.750 (0.326, 1.174)
Hosptial_3	0.550 (0.471, 0.629)	0.442 (0.439, 0.444)	0.892 (0.817, 0.968)	0.265 (0.198, 0.332)	0.322 (0.254, 0.390)	0.863 (0.768, 0.957)
Hosptial_4	0.519 (0.449, 0.588)	0.556 (0.555, 0.558)	0.482 (0.391, 0.574)	0.606 (0.532, 0.679)	0.451 (0.363, 0.539)	0.636 (0.562, 0.710)
ISUP_3–5 (*N* = 329)						
Hospital_2	0.512 (0.214, 0.811)	0.667 (0.642, 0.691)	0.375 (0.040, 0.710)	0.900 (0.714, 1.000)	0.750 (0.326, 1.174)	0.643 (0.392, 0.894)
Hosptial_3	0.617 (0.542, 0.691)	0.558 (0.556, 0.561)	0.455 (0.374, 0.536)	0.733 (0.639, 0.826)	0.742 (0.651, 0.833)	0.444 (0.362, 0.525)
Hosptial_4	0.557 (0.490, 0.624)	0.563 (0.562, 0.565)	0.916 (0.871, 0.962)	0.206 (0.139, 0.272)	0.539 (0.476, 0.602)	0.707 (0.568, 0.847)
Deep-radiomics model						
ISUP_2 (*N* = 152)						
Hospital_2	0.725 (0.474, 0.976)	0.722 (0.700, 0.744)	0.900 (0.714, 1.000)	0.500 (0.154, 0.846)	0.692 (0.441, 0.943)	0.800 (0.449, 1.151)
Hosptial_3	0.720 (0.651, 0.789)	0.632 (0.630, 0.634)	0.769 (0.667, 0.872)	0.578 (0.503, 0.653)	0.417 (0.328, 0.505)	0.865 (0.801, 0.928)
Hosptial_4	0.696 (0.635, 0.756)	0.616 (0.615, 0.618)	0.746 (0.666, 0.826)	0.529 (0.454, 0.604)	0.515 (0.439, 0.591)	0.756 (0.679, 0.833)
ISUP_3–5 (*N* = 329)						
Hospital_2	0.713 (0.461, 0.964)	0.667 (0.642, 0.691)	0.875 (0.646, 1.000)	0.500 (0.190, 0.810)	0.583 (0.304, 0.862)	0.833 (0.535, 1.132)
Hosptial_3	0.770 (0.709, 0.831)	0.654 (0.652, 0.656)	0.545 (0.464, 0.626)	0.837 (0.759, 0.915)	0.849 (0.777, 0.922)	0.522 (0.438, 0.605)
Hosptial_4	0.739 (0.683, 0.796)	0.665 (0.664, 0.667)	0.874 (0.820, 0.928)	0.454 (0.372, 0.536)	0.619 (0.552, 0.686)	0.780 (0.691, 0.870)
Biopsy pathology						
ISUP_2 (*N* = 152)						
Hospital_2	0.588 (0.394, 0.781)	0.556 (0.529, 0.582)	0.300 (0.016, 0.584)	0.875 (0.646, 1.000)	0.750 (0.326, 1.174)	0.500 (0.238, 0.762)
Hosptial_3	0.527 (0.483, 0.571)	0.706 (0.704, 0.707)	0.123 (0.043, 0.203)	0.934 (0.896, 0.972)	0.421 (0.199, 0.643)	0.731 (0.671, 0.791)
Hosptial_4	0.615 (0.555, 0.674)	0.630 (0.629, 0.632)	0.535 (0.444, 0.627)	0.694 (0.625, 0.763)	0.540 (0.448, 0.632)	0.690 (0.621, 0.759)
ISUP_3–5 (*N* = 329)						
Hospital_2	0.587 (0.394, 0.781)	0.556 (0.529, 0.582)	0.875 (0.646, 1.000)	0.300 (0.016, 0.584)	0.500 (0.238, 0.762)	0.750 (0.326, 1.174)
Hosptial_3	0.536 (0.496, 0.576)	0.641 (0.639, 0.643)	0.945 (0.908, 0.982)	0.128 (0.057, 0.198)	0.646 (0.582, 0.711)	0.579 (0.357, 0.801)
Hosptial_4	0.598 (0.541, 0.656)	0.592 (0.590, 0.593)	0.692 (0.617, 0.768)	0.489 (0.407, 0.572)	0.579 (0.505, 0.653)	0.611 (0.521, 0.701)

In Table 4, evaluation metrics, including AUC, accuracy (ACC), sensitivity (SEN), specificity (SPE), positive predictive value (PPV), negative predictive value (NPV), positive likelihood ratio (PLR), and negative likelihood ratio (NLR), are presented. In the external validation dataset, the AUCs for the prediction methods ranged from 0.597 to 0.718 for the clinical-imaging model, 0.554 to 0.613 for the PIRADS score, 0.566 to 0.632 for the radiomics model, 0.700 to 0.791 for the deep-radiomics model, and 0.537 to 0.578 for the biopsy. The ROC curves of the training dataset and the external validation dataset are depicted in Fig. 5.

For the prediction of ISUP_1, the AUC of the deep-radiomics model was the highest, surpassing those of the radiomics model, PIRADS score, and biopsy pathology (all *p* < 0.05); however, the deep-radiomics model did not significantly differ from the clinical-imaging model (*p *= 0.120). For the classification of ISUP_2, the AUC of the deep-radiomics model was the highest compared to those of the other prediction methods (all *p* < 0.05). For the classification of ISUP_3–5, the AUC of the deep-radiomics model also outperformed those of the other prediction methods (all *p* < 0.05). The results of the DeLong test for the comparison of AUCs in discriminating ISUP classes in the external validation dataset are shown in the supplementary material (Table S19).

Table 5 displays the AUCs evaluated across the three hospitals in the external dataset. The AUCs were compared using the Delong test, and the results are outlined in the supplementary material (Table S20). Since there was no ISUP_1 patient in the data from Hospital_2, a comparison of ISUP_1 could not be performed. For the prediction of ISUP_2, only the AUC of biopsy pathology was significantly different between Hospital_3 and Hospital_4 (*p* = 0.021). For the prediction of ISUP_3–5, only the AUC of the clinical-imaging model showed a statistically significant difference between Hospital_3 and Hospital_4 (*p* = 0.008). The PIRADS category, radiomics model, and deep-radiomics model did not significantly differ among the three hospitals in terms of the prediction of the ISUP_2 and ISUP_3–5 classes (all *p* > 0.05). The ROC curves for the predictions are illustrated in Fig. 6.

## Discussion

In this study, according to the International Society of Urology (ISUP) grading system, we divided the data into the ISUP 1, ISUP 2, and ISUP 3–5 groups, developed five methods to predict the grouping of PCa ISUP, and evaluated and verified the clinical efficacy of various prediction methods on external multi-center datasets. Our results showed that, compared with other methods, the radiomics model based on deep learning showed the best performance in predicting the invasiveness of PCa (the AUC ranged from 0.700 to 0.791), and there was no statistically significant difference among the other three external validation datasets (all *p* > 0.05). Our study showed that the radiomics model based on deep learning is promising for providing an objective and non-invasive method for evaluating the invasiveness of PCa, which is helpful for further reducing overtreatment and avoiding unnecessary biopsy and has a certain generalizability.

BpMRI can provide important information about the invasiveness of PCa [21, 22], and according to previous studies, ADC value had a high value in predicting GS [23, 24]. ADC value is calculated on the basis of DWI, which uses the diffusion attenuation of water molecules to reflect the microstructure of living tissues and has good reliability and stability [25]. The combination of radiomics and ADC in the diagnosis and evaluation of PCa has also been a research hotspot. Several researchers [26] have reported that the accuracy of feature prediction PCa of ADC sequence images can reach 97.39%, which means that better accuracy can be achieved only by extracting image features on ADC maps for prediction. Algohary [27] et al reported that intra-tumoral radiomics features on dual parameter MRI can predict the risk of PCa, and the radiomics features are mainly derived from ADC maps.

In this study, ADC maps were selected for feature extraction, and two models, traditional radiomics and deep-learning radiomics, were developed to evaluate their clinical application value in predicting the invasiveness of PCa. The results of this study showed that the AUC of the traditional radiomics model in the training cohort was 0.609 to 0.882, and the AUC of the three external validation datasets was only 0.566 to 0.632. Several previous single-center studies reported that [19, 28–30] the traditional radiomics model performed well in predicting the invasiveness of PCa (AUC value ranged from 0.70 to 0.93); however, this multi-centers study showed low accuracy. The reason may be that there is currently no unified machine-learning algorithm for traditional radiomics. The systematic and strict machine-learning framework includes feature extraction, classification, cross-validation, algorithm selection, and statistical analysis. The manual selection of these algorithm steps can affect the efficiency of the model to a certain extent [31]. Various algorithms have both advantages and disadvantages, and there is currently no unified standard for these algorithms. Improving the reproducibility of traditional radiomics models in different datasets still requires a large number of sample sets for research and validation.

Moreover, the deep-learning radiomics model showed good efficacy in this study. The model included 13 features extracted from the convolutional network (Table 5), and its prediction effect ranged from 0.791 to 0.882 in the training cohort and from 0.700 to 0.791 in the external verification cohort. The use of deep-learning algorithms is an important branch of machine learning. Various machine-learning algorithms are used on neural networks to learn the internal laws of sample data. Convolutional neural networks (CNNs) are the most representative models in deep learning. Many neural networks with different structures, such as AlexNet, GoogLeNet, ResNet, and DeepLab, have been created. These algorithms can more fully extract data features, thus realizing the algorithm set of various tasks [32]. Our research results were consistent with previous work. Similarly, Khosravi et al [33] developed a model based on GoogLeNet; their research showed that the AUC of the lesions that differentiated between the GG = 1 group and the GG ≥ 2 group was 0.78, while that of lesions that differentiated between the GG ≥ 4 group and the GG = 1 group was 0.86. Aldoj et al [34] conducted an analysis based on a multi-channel 3D CNN and used the open-source dataset ProstateX to establish a PCa classifier. Research has shown that the sensitivity, specificity, and AUC of the lesions in the GG ≥ 2 group and GG = 1 group were 81.9%, 86.1%, and 0.897, respectively. These results confirmed that deep-learning technology can mine high-dimensional features of images, has obvious advantages in building prediction models, provides a new idea for predicting the invasiveness of PCa, and is expected to be applied in the clinical environment in the future.

In recent years, although many studies have reported the broad prospects of the application of the radiomics model in PCa [13, 35, 36], few studies based on multi-center data or focused on invasion have been conducted. Multiple centers of data research verification are highly important for training the applicability and stability of the radiomics model and can promote its clinical application. In this study, there was no statistically significant difference in the prediction of ISUP grouping of PCa lesions by the in-depth learning radiomics model among the three external hospitals (*p* > 0.05), indicating that the model has strong generalizability among different hospitals, different MRI scanners, different field strengths, and different scanning parameters. The reasons may be as follows: (1) This study used a pre-trained AI algorithm to process bpMRI images for focus segmentation and feature extraction, which made the selection of ROIs more accurate and stable and reduced human error. (2) The ISUP groups included in the study all had postoperative RP pathology. Compared with the results of biopsy pathology [13], the pathological information in this study was more accurate. Previous studies have shown that the consistency of biopsy pathology and total prostatectomy pathology is only 54% [37]. (3) In this study, the radiomics model selected lesions and extracted features based on ADC maps. According to previous reports [38], there is no statistically significant difference (*p* = 0.51) in the stability of features extracted from different vendors’ scanners and ADC maps with different field strengths, which helps to develop a stable radiomics model and promote clinical application across multiple centers.

Our study has several limitations. Although this was a multi-center study, our cohort size was relatively small, and the distribution of patients and ISUP grouping data at each center were unbalanced, which may have affected our findings. Second, this study did not group the PCa in the TZ or PZ. Related studies have reported that [39] the effective radiomics features for PCa detection and evaluation differ between the TZ and PZs. However, in this study’s dataset, a significant number of lesions were large enough to span both the PZ and TZ, a scenario that is observed in 35.1% to 74.5% of cases across various datasets. If the analysis were to include only lesions confined to either the TZ or PZ, these cases would be ineligible for inclusion, which would significantly reduce the sample size available for research. To address this issue, future studies should aim to collect more cases, specifically those that are confined to either the PZ or the TZ. Incorporating the location of these lesions as a predictive factor in the model could potentially yield a more accurate model. By categorizing lesions based on their location in the TZ or PZ, future studies can extract radiomics features more accurately, which could subsequently improve the diagnostic efficiency of the model.

This multi-center study confirmed that the deep-learning radiomics model using AI to extract image features from bpMRI images can potentially predict the invasiveness of PCa and has strong external generalizability; additionally, this model is expected to provide an objective and a non-invasive new method for evaluating PCa in the clinic.

## Supplementary information

ELECTRONIC SUPPLEMENTARY MATERIAL

## Data Availability

The datasets used and analyzed during the current study are available from the corresponding author upon reasonable request.

## Abbreviations

ADC: Apparent diffusion coefficient
AI: Artificial intelligence
AUC: Area under the curve
bpMRI: Biparametric magnetic resonance imaging
DWI: Diffusion-weighted imaging
GG: Gleason grade group
GS: Gleason score
ISUP: International Society of Urology Pathology
mpMRI: Multiparametric magnetic resonance imaging
PCa: Prostate cancer
PZ: Peripheral zone
ROC: Receiver operating characteristic
ROI: Region of interest
RP: Radical prostatectomy
T2WI: T2-weighted imaging
TZ: Transitional zone
