# Supplementary material for: Evaluating the feasibility of AI-predicted bpMRI image features for predicting prostate cancer aggressiveness: a multi-center study
Source: Insights Imaging. 2025 Jan 15;16:20. doi: 10.1186/s13244-024-01865-8 (PMC11735704; doi:10.1186/s13244-024-01865-8)
Supplement: Supplementary file 1 — ELECTRONIC SUPPLEMENTARY MATERIAL [file 13244_2024_1865_MOESM1_ESM.pdf]

Evaluating the Feasibility of AI-Predicted bpMRI Image Features for Predicting Prostate Cancer Aggressiveness: a Multicenter Study

ELECTRONIC SUPPLEMENTARY MATERIAL

In our preestablished AI pipeline, we incorporate four separate AI models dedicated to tasks such as classifying MRI sequences, segmenting and measuring the prostate gland, identifying the zonal anatomy of the prostate, and detecting prostate cancer. Below, we provide specific descriptions for each of these AI models.

Model 1：MRI sequence classification

Data enrollment

A total of 1,153 mpMRI examinations taken from 1,086 patients between July 28, 2009, and Nov. 26, 2021, were retrospectively gathered. Following the import of anonymized data, DICOM files underwent conversion to Nifty format using dicom2nii.py in Python 3.5 to obtain the image data.

The DICOM data were initially divided into multiple scan sequences per MR examination, with individual sequences containing more than 15 slices being considered for the study.

Each sequence was then further subdivided into image groups based on matching acquisition parameters and spatial location. Diffusion weighted imaging (DWI) sequences were grouped by their b-value; for instance, a DWI sequence with 3 b-values was split into 3 separate image groups, each with a unique b-value.

Ultimately, a total of 5,151 images from five distinct types were classified. These included 1) DWI_High (b value ≥ 500 s/mm², n=1045), 2) DWI_Low (b value ≤ 100 s/mm², n=1012), 3) apparent diffusion coefficient (ADC) map (n=906), 4) T2-weighted imaging_nan (T2WI_nan) (nonfat-sat T2WI, n=1000), and 5) T2WI_fs (fat-sat T2WI, n=1188). T1-weighted imaging (T1WI) and dynamic contrast-enhanced (DCE) images were acquired but not included in the study.

MR scanners and imaging protocols

The mpMRI images were obtained from a total of 15 MR scanners from four different vendors. The transmit coils employed were body coils, while the receive coils utilized were phased array coils. No endorectal coil was utilized in this process. Further details regarding the MR scanners and image types can be found in Table S1.

Development of the deep learning model

The input image utilized an automatic window width and window level. Histogram equalization was applied, followed by resizing each image to 64×128×128 pixels. The training and validation datasets underwent augmentation through various image transformations, including rotation within a range of -10° to 10°, addition of random noise, perspective transformation, and translation by 0.01 pixels in cardinal or ordinal directions.

A total of 5,151 MR sequences were randomly divided into sets: 80% for training, 10% for validation, and 10% for testing. The modified Med3D network (refer to Figure S1) was retrained for classifying prostate mpMRI sequences. Utilizing transfer learning, we employed the encoder weights to extract image features. The encoder section was retained, while the decoder section (deconvolution part) was replaced with the convolution and fully connected layers of a conventional classification network structure. The classification convolution layer comprised four layers: 1) a max-pooling layer (stride: 2); 2) a convolution layer (kernel: 3); 3) another max-pooling layer (stride: 2); and 4) another convolution layer (kernel: 3). The fully connected layer of the classification network consisted of 128 neurons, where the image features were amalgamated and classified. The classification array was computed and output using the softmax function.

All training processes were executed on an NVIDIA Tesla P100 16G GPU. The algorithm was implemented in Python 3.6, utilizing PyTorch 0.4.1, OpenCV 3.4.0.12, Numpy 1.16.2, and SimpleITK 1.2.0. Classification efficiency was assessed using the confusion matrix. The detailed parameters of the deep learning training configuration file are presented in Table S3.

Results

The confusion matrix depicting the prediction results across various datasets can be found in Figure S2. Additionally, the corresponding prediction accuracies for the image classification model in different datasets are provided in Table S2. Specifically, the prediction accuracies for the training, validation, and testing datasets ranged between 99.2% and 100.0%, 98.9% and 100.0%, and 99.5% and 100.0%, respectively.

Model 2: Prostate gland segmentation and measurement

Data enrollment

The mpMRI images were obtained retrospectively from a cohort of 2,673 patients, involving a total of 2,849 mpMRI examinations conducted between July 28, 2009, and Nov. 26, 2021.

Following the import of anonymized data, the DICOM files underwent conversion to Nifty format using dicom2nii.py in Python 3.5. ADC maps (n=2320) were derived from the DWI sequence with both high and low b-values. Additionally, conventional T2WI and fat saturation T2WI (n=3654) were selected for further analysis.

MR scanners and imaging protocols

The mpMRI images were acquired from a total of 19 MR scanners sourced from four different vendors. The transmit coils employed were body coils, while the receive coils utilized were phased array coils. Further details regarding the MR scanners and image types can be found in Table S4.

Development of the deep learning model

The ground truth for the prostate gland was manually outlined by two experts with over 5 years of experience. The ADC and T2WI images were resized to 64×256×224 (z, y, x) pixels and used as input for the network. Data in the training set were augmented by applying random rotation (within 10°), adding random noise, and performing parallel translation within a range of [(-0.1; 0.1); (-0.1; 0.1)] pixels.

In a preliminary experiment (1), the classic U-Net (2) framework was employed. The U-Net model consists of an encoder-decoder structure with skip connections. The encoder portion is responsible for extracting features from the input image through a series of downsampling operations using convolutional layers. The decoder, on the other hand, upsamples the features back to the original image resolution through a series of upsampling operations. We downloaded the code of the U-Net model (https://github.com/milesial/Pytorch-UNet/tree/master) for the image segmentation task. The model training

was conducted on a personal computer with an Intel Core i5 3.2 GHz CPU, 16 GB of main memory, and an NVIDIA GTX1060 GPU. The detailed parameters of the deep learning training configuration file are presented in Table S5.

Results

The Dice similarity coefficient (DSC), Jaccard index, volumetric similarity (VS), Hausdorff distance (HD), and average distance (AD) were used to compare the model and manual segmentation results. The right and left (RL) diameter, anterior and posterior (AP) diameter, and superior and inferior (SI) diameter of the prostate gland were automatically measured using the algorithm rule of the minimum volume bounding box (Figure S3).

The DSC, Jaccard index, VS, HD, and AD in different datasets are shown in

Table S5 Parameters for training of Model 2

Configuration		Parameter
Input to the model		
	image	label of the prostate gland
Normalization of the signal intensity		
	low range	0
	high range	65535
Preprocess of the image		
	resample size	64:256:224 (z:y:x)
	crop of the image	(0, 1): (0.25, 0.9): (0.25, 0.75) (z:y:x)
Deep learning architecture		
	model name	Unet3D
	n_filters	16
Hyper parameter		
	batch size	3
	image size	64:256:224 (z:y:x)
	num epochs	400
	learning rate	0.001
Image augmentation		
	rotation	(-10, 10)
	noise	0.0001
	affine transformation	(0, 5)
	tilt	(0, 5)
	shift	(-0.01, 0.01), (-0.01, 0.01)
Postprocess of the output		

the maximum connected domain

Table S and Figure S4. Bland–Altman analysis of the measured values of the prostate gland, including RL diameter, AP diameter, SI diameter, volume, and signal intensity, are shown in

Table **S** and Figure S5. The differences between the manual label and the predicted label to their means were -2.058% to 4.257%.

Model 3: Prostate zonal anatomy segmentation

Prostate sextant locations model

First, the prostate gland is segmented by the established model (refer to Model 2). For sextant location, the prostatic gland was then trisected to obtain the base, mid-gland, and apex in the longitudinal axis direction. It was bisected to divide the prostate gland into left and right parts in the horizontal axis direction. Thus, the sextants were automatically generated (Figure S6).

Prostate zonal anatomy segmentation

Second, for the anatomic zone locations, we developed an anatomic regional model to segment the peripheral zone (PZ), transition zone (TZ), central zone (CZ), anterior fibromuscular stroma (AFS), urethra (URE), left seminal vesicle (LS) and right seminal vesicle (RS) (Figure S7).

Data enrollment

The mpMRI images were gathered retrospectively from a total of 1,225 patients, spanning from August 29, 2012, to November 26, 2021. Subsequent to importing the anonymized data, the DICOM files underwent conversion to Nifty format using dicom2nii.py in Python 3.5. T2WI was utilized for the creation of the prostate zonal anatomy segmentation model.

MR scanners and imaging protocols

T2WI images were acquired from a total of 17 MR scanners provided by four different vendors. The transmit coils utilized were body coils, while the receive coils employed were phased array coils. Detailed information regarding the MR scanning protocols can be found in Table S8.

Development of the deep learning model

T2WI images were resized to dimensions of 64×256×224 (z, y, x) pixels and served as the input for the network. Data augmentation in the training set involved random rotation (within a range of 10°), addition of random noise, and parallel translation within a range of [(-0.1; 0.1); (-0.1; 0.1)] pixels.

A total of 1,225 images were randomly divided into sets: 80% for training, 10% for validation, and 10% for testing. For prostate anatomic segmentation, a 3D U-Net segmentation framework (2) was employed. All training processes were carried out using the NVIDIA Tesla P100 16G GPU. The algorithm was implemented in Python 3.6, utilizing PyTorch 0.4.1, OpenCV 3.4.0.12, Numpy 1.16.2, and SimpleITK 1.2.0. The batch size was set to 10, and the networks were trained for a total of 300 epochs. The optimizer used was Adam, with a learning rate of 0.0001 and a binary cross-entropy loss function to minimize loss. The detailed parameters of the deep learning training configuration file are presented in Table S9.

Result

The DSC, JACRD, VS, HD, and AD in different datasets are shown in Table S10.

Model 4: Prostate cancer segmentation

Data enrollment

A total of 2,221 MRI images acquired between January 2014 and December 2019 were gathered retrospectively for the purpose of training an AI model for PCa segmentation. The inclusion criteria were specifically defined as follows: (a) a series of patients who underwent mpMRI prior to biopsy, primarily due to clinical suspicion of PCa arising from elevated serum prostate-specific antigen (PSA) levels, abnormal findings during digital rectal examination, and/or abnormal transrectal ultrasound results; (b) patients who subsequently underwent image-guided biopsy, transurethral prostatectomy, or radical prostatectomy within one month following the MRI examination and obtained pathological confirmation; (c) patients who had not received any PCa-related treatment prior to the examination; and (d) patients who tested negative for PCa during biopsy and exhibited no potential signs of PCa during clinical follow-up for a period exceeding one year.

The exclusion criteria encompassed (a) incomplete image data, (b)

substandard image quality, and (c) discrepancies between the MRI image and the pathology result, including variances in tumor location, clinically significant PCa (csPCa) not discernible through MRI, and cases where imaging suggested csPCa but pathology confirmed its absence.

MR scanners and imaging protocols

Prostate MRI images were obtained from a total of 16 different MR scanners to compile the model development dataset. The MRI sequences encompassed T1WI, T2WI, DWI, and ADC maps. Detailed information regarding the MR scanning protocols is presented in Table S11. The scanning parameters of DWI/ADC is presented in Table S12.

Development of the deep learning model

All included patients underwent TRUS-guided systematic (12- or 6-core needles) and cognitive-targeted biopsy (combined biopsy). Based on structured reports prepared by 1 of 5 dedicated urogenital radiologists with varying seniority (12, 16, 20, 22, and 28 years, respectively), lesions suspected of malignancy were marked on a prostate sector map for cognitive-targeted biopsy during the clinical routine. At least 1 urologist and 1 urogenital radiologist reviewed MR images before biopsy in a multidisciplinary meeting to ensure accurate localization of suspicious lesions. When performing cognitive-targeted biopsies, the urologists examined each suspicious lesion with an additional needle core (2 to 5-core needles). A urogenital pathologist with 11 years of experience conducted the histopathology analysis on each specimen. The pathology of systematic lesions and targeted lesions were fused into a combination reference. Those sextants were given the highest International Society of Urological Pathology (ISUP) grade as determined by either systematic biopsy or cognitive-targeted biopsy, whereas all other sextants were given a systematic ISUP grade. The csPCa was defined as ISUP grade 2 or higher based on histopathology findings and scored as Gleason score 3 + 4 or higher.

Two urogenital radiologists (Z.S. with 6 years of experience and X.W. with 30 years of experience) retrospectively reviewed all cases and mapped the pathology results of each focus to the MR images in consensus. If there was whole-mount step section pathology, then that was the reference standard; if not, the combined biopsy was the reference standard. Each lesion was manually delineated by the urogenital radiologist (Z.S.) and then modified by the other urogenital radiologist (X.W.) using the software ITK-SNAP. The modified annotations were used as ground truth.

To ensure accurate alignment, the DWI, ADC maps, and T2WI images were registered through rigid transformation using the coordinate information stored in the DICOM image headers. Standardization and cropping were applied to all prostate areas, resulting in a uniform size of 64×64×64 (x, y, z), with pixel intensities normalized within the range of [0, 1]. To augment the training set, random rotations (within a range of 10°), random noise, and parallel translations within the range of [(-0.1, 0.1); (-0.1, 0.1)] pixels were applied.

The dataset was randomly divided into three sets: 80% for training, 10% for validation, and 10% for internal testing. The AI model utilized a combination of DWI and ADC maps as input for PCa segmentation. For the segmentation process, a cascade 3D U-Net framework was employed. Model training was conducted on an NVIDIA Tesla P100 16G GPU, with the algorithm implemented using Python 3.6, PyTorch 0.4.1, OpenCV 3.4.0.12, Numpy 1.16.2, and SimpleITK 1.2.0. The detailed parameters of the deep learning training configuration file are presented in Table S13.

Result

In the test set, the model demonstrated excellent performance in lesion segmentation, achieving a DSC value of 0.80 [0.61, 0.86]. Additional evaluation metrics, including the Jaccard Index (JACRD), VS, HD, and AD in different datasets, can be found in Table S14.

Furthermore, Table S15 provides the measured values of PCa foci, encompassing the right-left (RL) diameter, anteroposterior (AP) diameter, superoinferior (SI) diameter, volume, and ADC value. Table S16 shows the Bland–Altman analysis comparing the AI-generated measurements with those annotated by radiologists, serving as the reference standard. These results indicate a high degree of agreement in the predicted volumes, three-dimensional diameters, and ADC values of prostate cancer lesions when compared to the reference standard. The observed differences between the majority of AI results and the reference standard fall within the 95% limit of agreement.

Execution order of the four AI models

In the actual clinical workflow, the execution order of the four AI models is as follows:

Step 1: Execute Model 1, which outputs DWI, ADC, and T2WI images. These images serve as inputs for Model 2.

Step 2: Execute Model 2, which outputs the prostate region. This output serves as input for both Model 3 and Model 4. Additionally, the prostate volume and diameter measurements obtained from Model 2 are automatically input into the structured report.

Step 3: Models 3 and 4 are executed in parallel.

Model 3 outputs the prostate's zonal regions.

Model 4 outputs the regions suspected of prostate cancer, and it automatically records the size and ADC value of the suspected cancer in the structured report under the "prostate cancer size" and "ADC value" sections.

Step 4: The results from Model 3 and Model 4 are integrated to provide the prostate cancer localization. This localization data is then automatically entered into the structured report under the "prostate cancer localization" section.

Below is the flowchart representing the model execution process:

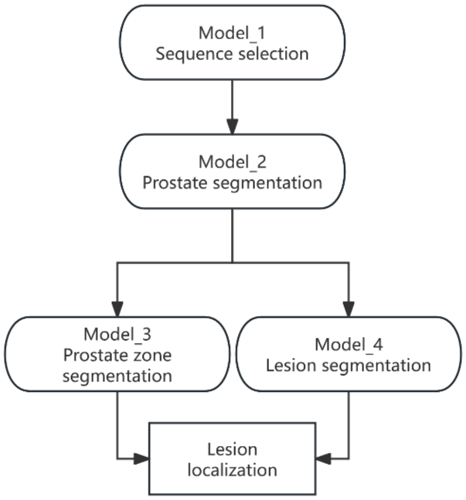

Studies about the proposed models

Over the years, we have been consistently testing and enhancing our AI models for prostate cancer detection, with several publications that have assessed performance at both lesion and patient levels. We have utilized metrics such as sensitivity, specificity, and the area under the receiver operating characteristic curve (AUC) to gauge the models' accuracy(1). Furthermore, the model's efficacy has been assessed using various external multicenter datasets, with an emphasis on its utility for radiologists. The pertinent details and extra references can be found in the supplementary material (refer to page x, lines x to x) (2-4).

Evaluation of the multiclass classification efficacy

The statistical analysis process for multiclass classification is as following:

1. We employed an ordinal multiclass logistic regression model to predict all cases in the dataset. Each case received a predicted probability.
2. Using the multiclass ROC analysis method(5), we determined the optimal cutoff value for the predicted probabilities of each class. Based on this optimal cutoff value, we classified each case and produced a three-class confusion matrix. The software tool used was the multiclass.roc() function from the pROC package in R (version 4.1.3).
3. Furthermore, based on the multiclass ROC analysis results, we calculated the diagnostic performance for multiclass classification, including AUC, accuracy, sensitivity, specificity, positive predictive value, and negative predictive value(6), as presented in Tables 4 and 5. The software tool used was the reportROC() function from the reportROC package in R (version 4.1.3).

Supplementary Tables

Table S1 Information on the MR scanners and the image types

	Overall (n=5151)	Training (n=4122)	Validation (n=513)	Testing (n=516)	*P* value
Age (yrs)					
Median [Q1, Q3]	71.0 [65.0, 76.0]	71.0 [65.0, 76.0]	71.0 [66.0, 77.0]	71.0 [65.0, 76.0]	0.46
Image Type					
ADC	906 (17.6%)	730 (17.7%)	86 (16.8%)	90 (17.4%)	>0.99
DWI_High	1045 (20.3%)	835 (20.3%)	105 (20.5%)	105 (20.3%)	
DWI_Low	1012 (19.6%)	808 (19.6%)	102 (19.9%)	102 (19.8%)	
T2WI_Fs	1188 (23.1%)	950 (23.0%)	120 (23.4%)	118 (22.9%)	
T2WI_nan	1000 (19.4%)	799 (19.4%)	100 (19.5%)	101 (19.6%)	
Magnetic Field					
1.5 T	657 (12.8%)	523 (12.7%)	59 (11.5%)	75 (14.5%)	0.33
3.0 T	4494 (87.2%)	3599 (87.3%)	454 (88.5%)	441 (85.5%)	
Manufacture					
GE Medical Systems	2635 (51.2%)	2100 (50.9%)	253 (49.3%)	282 (54.7%)	0.50
Philips Medical Systems	491 (9.5%)	397 (9.6%)	50 (9.7%)	44 (8.5%)	
SIEMENS	2025	1625	210	190 (36.8%)	

	(39.3%)	(39.4%)	(40.9%)		
Station Name					
AWP145938	597 (11.6%)	468 (11.4%)	73 (14.2%)	56 (10.9%)	0.17
AWP152194	119 (2.3%)	96 (2.3%)	12 (2.3%)	11 (2.1%)	
AWP166059	194 (3.8%)	164 (4.0%)	17 (3.3%)	13 (2.5%)	
AWP174090	8 (0.2%)	5 (0.1%)	0 (0%)	3 (0.6%)	
AWP39300	6 (0.1%)	5 (0.1%)	0 (0%)	1 (0.2%)	
DVMRDVMR	1172 (22.8%)	939 (22.8%)	124 (24.2%)	109 (21.1%)	
GEHC	1023 (19.9%)	821 (19.9%)	87 (17.0%)	115 (22.3%)	
GEHCGEHC	440 (8.5%)	340 (8.2%)	42 (8.2%)	58 (11.2%)	
MRC35207	696 (13.5%)	567 (13.8%)	69 (13.5%)	60 (11.6%)	
MRC40764	387 (7.5%)	306 (7.4%)	37 (7.2%)	44 (8.5%)	
MRSUZTB03A	57 (1.1%)	49 (1.2%)	4 (0.8%)	4 (0.8%)	
PHILIPS-8FA1B4E	72 (1.4%)	62 (1.5%)	5 (1.0%)	5 (1.0%)	
PHILIPS-CB0GKAC	12 (0.2%)	9 (0.2%)	0 (0%)	3 (0.6%)	
PHILIPS-DSALI1J	156 (3.0%)	124 (3.0%)	17 (3.3%)	15 (2.9%)	
PHILIPS-NK6RG9A	194 (3.8%)	153 (3.7%)	24 (4.7%)	17 (3.3%)	

Quantitative variables are given as the median [Q1, Q3] for nonnormalized data.

Fs fat saturation, T2WI T2-weighted imaging, ADC apparent diffusion coefficient, DWI diffusion-weighted imaging.

Table S2 Prediction efficacies of the image classification model in different datasets

	Image Type	Image Number	Accuracy	Sensitivity	Specificity	PPV	NPV	F1	Kappa	Prevalence	Detection Rate	Detection Prevalence
Train												
	ADC	718	1.000	1.000	1.000	0.999	1.000	0.999	0.999	0.174	0.174	0.174
	DWI_High	849	0.996	0.994	0.998	0.991	0.998	0.992	0.990	0.206	0.205	0.207
	DWI_Low	815	0.992	0.987	0.998	0.991	0.997	0.989	0.986	0.198	0.195	0.197
	T2WI_Fs	957	0.998	0.997	0.999	0.998	0.999	0.997	0.997	0.232	0.231	0.232
	T2WI_nan	783	1.000	1.000	1.000	0.999	1.000	0.999	0.999	0.190	0.190	0.190
Validation												
	ADC	96	1.000	1.000	1.000	1.000	1.000	1.000	1.000	0.187	0.187	0.187
	DWI_High	93	0.989	0.978	1.000	1.000	0.995	0.989	0.987	0.181	0.177	0.177
	DWI_Low	101	0.998	1.000	0.995	0.981	1.000	0.990	0.988	0.197	0.197	0.201
	T2WI_Fs	107	1.000	1.000	1.000	1.000	1.000	1.000	1.000	0.209	0.209	0.209
	T2WI_nan	116	1.000	1.000	1.000	1.000	1.000	1.000	1.000	0.226	0.226	0.226
Test												
	ADC	92	1.000	1.000	1.000	1.000	1.000	1.000	1.000	0.178	0.178	0.178
	DWI_High	103	0.995	0.990	1.000	1.000	0.998	0.995	0.994	0.200	0.198	0.198
	DWI_Low	96	0.999	1.000	0.998	0.990	1.000	0.995	0.994	0.186	0.186	0.188
	T2WI_Fs	124	1.000	1.000	1.000	1.000	1.000	1.000	1.000	0.240	0.240	0.240
	T2WI_nan	101	1.000	1.000	1.000	1.000	1.000	1.000	1.000	0.196	0.196	0.196

ADC apparent diffusion coefficient, T2WI T2-weighted imaging, DWI diffusion weighted imaging, Fs fat saturation, PPV positive predictive value, NPV negative predictive value.

Table S3 Parameters for training of Model 1

Configuration		Parameter
Input to the model		
	image series	5 classes
Normalization of the signal intensity		
	low range	0
	high range	65535
Preprocess of the image		
	resample size	64:128:128 (z:y:x)
Deep learning architecture		
	model name	Med3D
	model depth	18
	pretrained	Yes
	conv cfg	M, 512, M, 512
	hidden layer cft	128
	dropout	Yes
Hyper parameter		
	batch size	40
	image size	64:128:128 (z:y:x)
	num epochs	400
	learning rate	0.001
Image augmentation		
	rotation	(-10, 10)
	noise	0.0001
	affine transformation	(0, 5)

	tilt	(0, 5)
	shift	(-0.01, 0.01), (-0.01, 0.01)
Postprocess of the output		
	predicted class with the maximum probability	

Table S4 Information on the MR scanners and the image types

	Overall (n=5974)	Train (n=4780)	Validation (n=601)	Test (n=593)	*P* Value
Magnetic Field					0.50
1.5T	1034 (17.3%)	841 (17.6%)	96 (16.0%)	97 (16.4%)	
3.0T	4940 (82.7%)	3939 (82.4%)	505 (84.0%)	496 (83.6%)	
Image Type					0.35
ADC	2320 (38.8%)	1873 (39.2%)	217 (36.1%)	230 (38.8%)	
T2WI	3654 (61.2%)	2907 (60.8%)	384 (63.9%)	363 (61.2%)	
Manufacture					0.79
GE MEDICAL SYSTEMS	3243 (54.3%)	2599 (54.4%)	316 (52.6%)	328 (55.3%)	
Philips Medical Systems	810 (13.6%)	637 (13.3%)	91 (15.1%)	82 (13.8%)	
SIEMENS	1695 (28.4%)	1368 (28.6%)	168 (28.0%)	159 (26.8%)	
UIH	226 (3.8%)	176 (3.7%)	26 (4.3%)	24 (4.0%)	
Model Name					0.87
Achieva	150 (2.5%)	123 (2.6%)	17 (2.8%)	10 (1.7%)	
Ingenia	583 (9.8%)	456 (9.5%)	64 (10.6%)	63 (10.6%)	
Ingenia CX	3 (0.1%)	2 (0.0%)	1 (0.2%)	0 (0%)	
Discovery MR750	2753 (46.1%)	2204 (46.1%)	276 (45.9%)	273 (46.0%)	
Discovery MR750w	304 (5.1%)	250 (5.2%)	21 (3.5%)	33 (5.6%)	
Signa EXCITE	173 (2.9%)	135 (2.8%)	16 (2.7%)	22 (3.7%)	
Signa HDxt	11 (0.2%)	9 (0.2%)	2 (0.3%)	0 (0%)	
Signa Premier	2 (0.0%)	1 (0.0%)	1 (0.2%)	0 (0%)	

Aera	889 (14.9%)	724 (15.1%)	81 (13.5%)	84 (14.2%)
Amira	4 (0.1%)	3 (0.1%)	1 (0.2%)	0 (0%)
Essenza	2 (0.0%)	2 (0.0%)	0 (0%)	0 (0%)
Multiva	74 (1.2%)	56 (1.2%)	9 (1.5%)	9 (1.5%)
Prisma	127 (2.1%)	99 (2.1%)	16 (2.7%)	12 (2.0%)
Skyra	188 (3.1%)	156 (3.3%)	18 (3.0%)	14 (2.4%)
TrioTim	348 (5.8%)	273 (5.7%)	39 (6.5%)	36 (6.1%)
Verio	137 (2.3%)	111 (2.3%)	13 (2.2%)	13 (2.2%)
uMR 790	226 (3.8%)	176 (3.7%)	26 (4.3%)	24 (4.0%)

Quantitative variables are given as the median [Q1, Q3] for nonnormalized data. ADC apparent diffusion coefficient, T2WI T2-weighted imaging

Table S5 Parameters for training of Model 2

Configuration		Parameter
Input to the model		
	image	label of the prostate gland
Normalization of the signal intensity		
	low range	0
	high range	65535
Preprocess of the image		
	resample size	64:256:224 (z:y:x)
	crop of the image	(0, 1): (0.25, 0.9): (0.25, 0.75) (z:y:x)
Deep learning architecture		
	model name	Unet3D
	n_filters	16
Hyper parameter		
	batch size	3
	image size	64:256:224 (z:y:x)
	num epochs	400

	learning rate	0.001
Image augmentation		
	rotation	(-10, 10)
	noise	0.0001
	affine transformation	(0, 5)
	tilt	(0, 5)
	shift	(-0.01, 0.01), (-0.01, 0.01)
Postprocess of the output		
	the maximum connected domain	

Table S6 Segmentation metrics in different datasets

	Overall		Train		Validation		Test	
	ADC (n=2320)	T2WI (n=3654)	ADC (n=1873)	T2WI (n=2907)	ADC (n=217)	T2WI (n=384)	ADC (n=230)	T2WI (n=363)
DSC	0.921 (0.0337)	0.937 (0.0322)	0.925 (0.0279)	0.940 (0.0270)	0.902 (0.0491)	0.922 (0.0445)	0.900 (0.0446)	0.923 (0.0449)
JACRD	0.854 (0.0549)	0.883 (0.0537)	0.862 (0.0467)	0.889 (0.0458)	0.825 (0.0755)	0.857 (0.0717)	0.822 (0.0704)	0.861 (0.0726)
VS	0.970 (0.0303)	0.979 (0.0245)	0.974 (0.0236)	0.981 (0.0200)	0.953 (0.0478)	0.967 (0.0370)	0.955 (0.0449)	0.971 (0.0339)
HD	6.460 (3.150)	5.880 (2.830)	6.20 (2.720)	5.640 (2.490)	7.450 (4.700)	6.780 (3.730)	7.700 (3.990)	6.840 (3.730)
AD	0.140 (0.149)	0.168 (4.01)	0.122 (0.0962)	0.172 (4.49)	0.213 (0.293)	0.152 (0.196)	0.214 (0.233)	0.149 (0.186)

Data conforming to a normal distribution are presented as the mean (standard deviation). DSC Dice similarity coefficient, VS volumetric similarity, HD Hausdorff distance, AD Average distance, T2WI T2-weighted imaging, ADC apparent diffusion coefficient.

Table S7 Bland–Altman analysis of the measured values of the prostate gland

	RL Diameter (mm)	AP Diameter (mm)	SI Diameter (mm)	Volume (cm^3)	Signal Intensity
Means of label and plabel	56.025	59.670	63.655	108.623	56.025
Differences	2.230	2.540	-1.310	-0.924	2.230
Means/differences proportion	3.980	4.257	-2.058	-0.851	3.980
Means of label	57.140	60.940	63.000	108.160	57.140
Means of plabel	54.910	58.400	64.310	109.085	54.910
Bias of the label and plabel	0.733	1.094	-1.180	-0.623	0.733
Bias Upper CI	0.800	1.172	-1.063	-0.477	0.800
Bias Lower CI	0.665	1.017	-1.298	-0.769	0.665
Bias Std Dev	2.679	3.057	4.643	5.756	2.679
Bias Standard Error	0.035	0.040	0.060	0.745	0.035
LOA Standard Error	0.059	0.068	0.103	0.127	0.059
Upper LOA	5.984	7.085	7.919	10.659	5.984
Upper LOA_upperCI	6.100	7.218	8.120	10.908	6.100
Upper LOA_lowerCI	5.868	6.953	7.718	10.409	5.868
Lower LOA	-4.519	-4.897	-10.280	-11.904	-4.519
Lower LOA_upperCI	-4.403	-4.764	-10.078	-11.655	-4.403
Lower LOA_lowerCI	-4.635	-5.029	-10.481	-12.154	-4.635
Regression fixed slope	0.076	0.071	0.032	0.023	0.076
Regression fixed intercept	-3.100	-2.100	-2.700	-1.900	-3.100

LOA limits of agreement, CI confidence interval, RL right and left, AP anterior and posterior, SI superior and inferior

Table S8 Scanning protocols of the T2WI

	Overall (N=1225)	train (N=973)	validate (N=99)	test (N=153)	*P* value
Magnetic Field					
1.5T	271 (22.1%)	213 (21.9%)	14 (14.1%)	44 (28.8%)	0.02
3.0T	954 (77.9%)	760 (78.1%)	85 (85.9%)	109 (71.2%)	
Manufacture					
GE MEDICAL SYSTEMS	665 (54.3%)	540 (55.5%)	65 (65.7%)	60 (39.2%)	<0.001
Philips Medical Systems	155 (12.7%)	111 (11.4%)	10 (10.1%)	34 (22.2%)	
SIEMENS	365 (29.8%)	289 (29.7%)	20 (20.2%)	56 (36.6%)	
UIH	40 (3.3%)	33 (3.4%)	4 (4.0%)	3 (2.0%)	
Model Name					
Achieva	26 (2.1%)	20 (2.1%)	3 (3.0%)	3 (2.0%)	0.01
Aera	239 (19.5%)	192 (19.7%)	8 (8.1%)	39 (25.5%)	
Amira	1 (0.1%)	1 (0.1%)	0 (0%)	0 (0%)	
DISCOVERY MR750	547 (44.7%)	446 (45.8%)	54 (54.5%)	47 (30.7%)	
DISCOVERY MR750w	78 (6.4%)	64 (6.6%)	5 (5.1%)	9 (5.9%)	
Ingenia	111 (9.1%)	79 (8.1%)	5 (5.1%)	27 (17.6%)	
Ingenia CX	1 (0.1%)	1 (0.1%)	0 (0%)	0 (0%)	
MAGNETOM_ESSENZA	1 (0.1%)	1 (0.1%)	0 (0%)	0 (0%)	
Multiva	17 (1.4%)	11 (1.1%)	2 (2.0%)	4 (2.6%)	
Prisma	10 (0.8%)	7 (0.7%)	1 (1.0%)	2 (1.3%)	

SIGNA EXCITE	36 (2.9%)	28 (2.9%)	5 (5.1%)	3 (2.0%)	
Signa HDxt	3 (0.2%)	1 (0.1%)	1 (1.0%)	1 (0.7%)	
SIGNA Premier	1 (0.1%)	1 (0.1%)	0 (0%)	0 (0%)	
Skyra	34 (2.8%)	28 (2.9%)	3 (3.0%)	3 (2.0%)	
TrioTim	41 (3.3%)	35 (3.6%)	2 (2.0%)	4 (2.6%)	
uMR 790	40 (3.3%)	33 (3.4%)	4 (4.0%)	3 (2.0%)	
Verio	39 (3.2%)	25 (2.6%)	6 (6.1%)	8 (5.2%)	
FatSat					
fs	87 (7.1%)	67 (6.9%)	10 (10.1%)	10 (6.5%)	0.47
nonfs	1138 (92.9%)	906 (93.1%)	89 (89.9%)	143 (93.5%)	
Repetition Time (ms)					
Median [Q1, Q3]	3560 [3040, 3880]	3460 [3040, 3850]	3560 [3070, 3790]	3730 [3000, 4200]	0.30
Echo Time (ms)					
Median [Q1, Q3]	92.9 [87.5, 112]	92.2 [87.4, 110]	90.3 [87.4, 103]	99.0 [88.0, 115]	0.05
Pixel Bandwidth (Hz)					
Median [Q1, Q3]	163 [163, 200]	163 [163, 200]	163 [122, 188]	200 [160, 218]	<0.001
Flip Angle					
Median [Q1, Q3]	111 [111, 140]	111 [111, 140]	111 [111, 111]	111 [111, 150]	0.37
Reconstruction Diameter					
Median [Q1, Q3]	240 [200, 240]	240 [200, 240]	240 [200, 240]	220 [200, 240]	0.01
Slice Thickness (mm)					

Median [Q1, Q3]	4.00 [3.50, 4.00]	4.00 [3.50, 4.00]	4.00 [3.40, 4.00]	4.00 [3.50, 4.00]	0.44
Slice Spacing (mm)					
Median [Q1, Q3]	4.00 [4.00, 4.00]	4.00 [4.00, 4.00]	4.00 [4.00, 4.00]	4.00 [3.60, 4.00]	0.06
Pixel Spacing (mm)					
Median [Q1, Q3]	0.469 [0.469, 0.577]	0.469 [0.469, 0.625]	0.469[0.417, 0.469]	0.469 [0.344, 0.625]	0.05

Table S9 Parameters for training of Model 3

Configuration		Parameter
Input to the model		
	image	label of the zonal areas of the prostate gland
Normalization of the signal intensity		
	low range	0
	high range	65535
Preprocess of the image		
	resample size	64:256:256 (z:y:x)
	crop of the image	mask of the prostate gland from Model 2
Deep learning architecture		
	model name	Unet3D
	n_filters	16
Hyper parameter		
	batch size	3
	image size	64:256:256 (z:y:x)
	num epochs	400
	learning rate	0.001

Image augmentation		
	rotation	(-10, 10)
	noise	0.0001
	affine transformation	(0, 5)
	tilt	(0, 5)
	shift	(-0.01, 0.01), (-0.01, 0.01)
Postprocess of the output		
	the maximum connected domain for each zonal area	

Table S10 Segmentation metrics of the model

	Overall (N=1225)	train (N=979)	validate (N=123)	test (N=123)	P Value
AFS					
DSC					
Median [Min, Max]	0.790 [0, 0.920]	0.800 [0, 0.920]	0.710 [0, 0.890]	0.690 [0.0300, 0.860]	<0.001
Missing	11 (0.9%)	8 (0.8%)	1 (0.8%)	2 (1.6%)	
JACRD					
Median [Min, Max]	0.650 [0, 0.850]	0.670 [0, 0.850]	0.550 [0, 0.800]	0.530 [0.020, 0.760]	<0.001
Missing	11 (0.9%)	8 (0.8%)	1 (0.8%)	2 (1.6%)	
VS					
Median [Min, Max]	0.930 [0.0300, 1.00]	0.940 [0.0300, 1.00]	0.885 [0.230, 1.00]	0.890 [0.140, 1.00]	<0.001
Missing	11 (0.9%)	8 (0.8%)	1 (0.8%)	2 (1.6%)	
HD					
Median [Min, Max]	5.05 [1.56, 50.3]	4.77 [1.56, 50.3]	6.89 [2.21, 47.0]	6.64 [2.50, 48.6]	<0.001
Missing	11 (0.9%)	8 (0.8%)	1 (0.8%)	2 (1.6%)	
AD					
Median [Min, Max]	0.260 [0.0900, 25.3]	0.230 [0.090, 25.3]	0.410 [0.100, 9.58]	0.450 [0.130, 3.73]	<0.001
Missing	11 (0.9%)	8 (0.8%)	1 (0.8%)	2 (1.6%)	
PZ					

DSC					
Median [Min, Max]	0.870 [0, 0.960]	0.88 [0, 0.96]	0.84 [0.48, 0.92]	0.840 [0.390, 0.930]	<0.001
Missing	1 (0.1%)	1 (0.1%)	0 (0%)	0 (0%)	
JACRD					
Median [Min, Max]	0.770 [0, 0.920]	0.780 [0, 0.920]	0.720 [0.31, 0.86]	0.720 [0.240, 0.870]	
Missing	1 (0.1%)	1 (0.1%)	0 (0%)	0 (0%)	
VS					
Median [Min, Max]	0.970 [0.100, 1.00]	0.970 [0.100, 1.00]	0.95 [0.61, 1.00]	0.960 [0.530, 1.00]	<0.001
Missing	1 (0.1%)	1 (0.1%)	0 (0%)	0 (0%)	
HD					
Median [Min, Max]	7.55 [2.50, 50.2]	7.20 [2.50, 43.7]	8.91 [2.58, 50.2]	8.11 [3.85, 44.3]	<0.001
Missing	1 (0.1%)	1 (0.1%)	0 (0%)	0 (0%)	
AD					
Median [Min, Max]	0.160 [0.0500, 19.5]	0.150 [0.0500, 19.5]	0.240 [0.080, 2.05]	0.240 [0.080, 4.43]	<0.001
Missing	1 (0.1%)	1 (0.1%)	0 (0%)	0 (0%)	
CZ					
DSC					
Median [Min, Max]	0.810 [0, 0.930]	0.820 [0.410, 0.930]	0.650 [0.05, 0.87]	0.630 [0, 0.900]	<0.001
Missing	6 (0.5%)	6 (0.6%)	0 (0%)	0 (0%)	
JACRD					
Median [Min, Max]	0.680 [0, 0.880]	0.700 [0.260, 0.880]	0.480 [0.03, 0.770]	0.460 [0, 0.810]	<0.001
Missing	6 (0.5%)	6 (0.6%)	0 (0%)	0 (0%)	
VS					

Median [Min, Max]	0.920 [0.170, 1.00]	0.930 [0.490, 1.00]	0.88 [0.180, 1.00]	0.880 [0.170, 1.00]	<0.001
Missing	6 (0.5%)	6 (0.6%)	0 (0%)	0 (0%)	
AD					
Median [Min, Max]	4.60 [2.00, 45.5]	4.29 [2.00, 34.1]	6.53 [2.80, 45.5]	6.50 [2.73, 33.0]	<0.001
Missing	6 (0.5%)	6 (0.6%)	0 (0%)	0 (0%)	
HD					
Median [Min, Max]	0.240 [0.0600, 9.09]	0.220 [0.060, 3.89]	0.600 [0.140, 8.41]	0.610 [0.120, 9.09]	<0.001
Missing	6 (0.5%)	6 (0.6%)	0 (0%)	0 (0%)	
TZ					
DSC					
Median [Min, Max]	0.930 [0.610, 0.970]	0.940 [0.720, 0.970]	0.910 [0.610, 0.970]	0.920 [0.700, 0.970]	<0.001
Missing	1 (0.1%)	1 (0.1%)	0 (0%)	0 (0%)	
JACRD					
Median [Min, Max]	0.870 [0.440, 0.950]	0.880 [0.560, 0.950]	0.830 [0.44, 0.940]	0.850 [0.540, 0.940]	<0.001
Missing	1 (0.1%)	1 (0.1%)	0 (0%)	0 (0%)	
VS					
Median [Min, Max]	0.980 [0.770, 1.00]	0.990 [0.840, 1.00]	0.970 [0.770, 1.00]	0.970 [0.830, 1.00]	<0.001
Missing	1 (0.1%)	1 (0.1%)	0 (0%)	0 (0%)	
HD					
Median [Min, Max]	4.59 [2.34, 38.3]	4.46 [2.34, 34.0]	5.33 [2.47, 38.3]	4.91 [2.72, 19.4]	<0.001
Missing	1 (0.1%)	1 (0.1%)	0 (0%)	0 (0%)	
AD					
Median [Min, Max]	0.080 [0.020,	0.080 [0.020,	0.130 [0.03,	0.110 [0.0300,	<0.001

	1.07]	0.890]	1.07]	0.730]	
Missing	1 (0.1%)	1 (0.1%)	0 (0%)	0 (0%)	
URE					
DSC					
Median [Min, Max]	0.910 [0, 0.980]	0.920 [0.520, 0.980]	0.830 [0, 0.960]	0.830 [0.490, 0.960]	<0.001
Missing	8 (0.7%)	5 (0.5%)	1 (0.8%)	2 (1.6%)	
JACRD					
Median [Min, Max]	0.830 [0, 0.960]	0.840 [0.350, 0.960]	0.700 [0, 0.930]	0.700 [0.320, 0.930]	<0.001
Missing	8 (0.7%)	5 (0.5%)	1 (0.8%)	2 (1.6%)	
VS					
Median [Min, Max]	0.940 [0.0800, 1.00]	0.950 [0.550, 1.00]	0.890 [0.080, 1.00]	0.900 [0.490, 1.00]	<0.001
Missing	8 (0.7%)	5 (0.5%)	1 (0.8%)	2 (1.6%)	
HD					
Median [Min, Max]	1.88 [0.780, 49.5]	1.75 [0.780, 49.5]	3.31 [0.940, 17.1]	3.13 [0.780, 33.8]	<0.001
Missing	8 (0.7%)	5 (0.5%)	1 (0.8%)	2 (1.6%)	
AD					
Median [Min, Max]	0.0900 [0.020, 723]	0.080 [0.020, 1.18]	0.220 [0.04, 723]	0.200 [0.03, 1.13]	<0.001
Missing	8 (0.7%)	5 (0.5%)	1 (0.8%)	2 (1.6%)	
RS					
DSC					
Median [Min, Max]	0.920 [0, 0.970]	0.930 [0, 0.970]	0.900 [0.710, 0.97]	0.900 [0, 0.970]	<0.001
Missing	1 (0.1%)	1 (0.1%)	0 (0%)	0 (0%)	

JACRD					
Median [Min, Max]	0.860 [0, 0.940]	0.860 [0, 0.940]	0.82 [0.550, 0.930]	0.830 [0, 0.940]	<0.001
Missing	1 (0.1%)	1 (0.1%)	0 (0%)	0 (0%)	
VS					
Median [Min, Max]	0.970 [0.760, 1.00]	0.980 [0.780, 1.00]	0.970 [0.760, 1.00]	0.960 [0.760, 1.00]	<0.001
Missing	1 (0.1%)	1 (0.1%)	0 (0%)	0 (0%)	
AD					
Median [Min, Max]	4.17 [1.37, 52.9]	3.98 [1.37, 52.9]	4.94 [1.92, 39.7]	4.74 [1.88, 37.0]	<0.001
Missing	1 (0.1%)	1 (0.1%)	0 (0%)	0 (0%)	
HD					
Median [Min, Max]	0.090 [0.030, 805]	0.080 [0.030, 805]	0.130 [0.03, 107]	0.120 [0.030, 15.1]	<0.001
Missing	1 (0.1%)	1 (0.1%)	0 (0%)	0 (0%)	
LS					
DSC					
Median [Min, Max]	0.920 [0.080, 0.970]	0.930 [0.260, 0.970]	0.90 [0.08, 0.960]	0.900 [0.260, 0.960]	<0.001
Missing	1 (0.1%)	1 (0.1%)	0 (0%)	0 (0%)	
JACRD					
Median [Min, Max]	0.860 [0.040, 0.950]	0.860 [0.150, 0.950]	0.830 [0.04, 0.920]	0.830 [0.150, 0.920]	<0.001
Missing	1 (0.1%)	1 (0.1%)	0 (0%)	0 (0%)	
VS					
Median [Min, Max]	0.980 [0.120, 1.00]	0.980 [0.800, 1.00]	0.970 [0.120, 1.00]	0.960 [0.260, 1.00]	<0.001
Missing	1 (0.1%)	1 (0.1%)	0 (0%)	0 (0%)	

HD					
Median [Min, Max]	3.75 [1.33, 42.3]	3.75 [1.33, 41.3]	4.26 [1.88, 35.5]	4.42 [2.08, 42.3]	<0.001
Missing	1 (0.1%)	1 (0.1%)	0 (0%)	0 (0%)	
AD					
Median [Min, Max]	0.0900 [0.030, 9.54]	0.080 [0.030, 9.54]	0.110 [0.04, 4.80]	0.130 [0.0400, 1.70]	<0.001
Missing	1 (0.1%)	1 (0.1%)	0 (0%)	0 (0%)	

Categorical variables are given as numbers (percentages). Quantitative variables are given as the median [Q1, Q3] for nonnormalized data. DSC Dice similarity coefficient, VS volumetric similarity, HD Hausdorff distance, AD Average distance, AFS anterior fibromuscular stroma, PZ peripheral zone, CZ central zone, TZ transition zone, URE urethra, LS left seminal vesicle, RS right seminal vesicle.

Table S11 Information on MR scanners and protocols

	Train (N=1681)	Validate (N=212)	Test (N=212)
**DWI/ADC**			
Model Name			
Achieva	52 (3.1%)	5 (2.4%)	4 (1.9%)
Aera	217 (12.9%)	32 (15.1%)	22 (10.4%)
Amira	1 (0.1%)	1 (0.5%)	0 (0%)
DISCOVERY MR750	844 (50.2%)	112 (52.8%)	109 (51.4%)
DISCOVERY MR750w	75 (4.5%)	13 (6.1%)	7 (3.3%)
Ingenia	107 (6.4%)	8 (3.8%)	19 (9.0%)
Ingenia CX	1 (0.1%)	0 (0%)	0 (0%)
Multiva	24 (1.4%)	2 (0.9%)	4 (1.9%)
Prisma	29 (1.7%)	2 (0.9%)	4 (1.9%)
SIGNA EXCITE	36 (2.1%)	2 (0.9%)	4 (1.9%)
Signa HDxt	3 (0.2%)	0 (0%)	0 (0%)
SIGNA Premier	1 (0.1%)	0 (0%)	0 (0%)
Skyra	46 (2.7%)	6 (2.8%)	8 (3.8%)
TrioTim	114 (6.8%)	17 (8.0%)	18 (8.5%)
uMR 790	87 (5.2%)	8 (3.8%)	7 (3.3%)
Verio	44 (2.6%)	4 (1.9%)	6 (2.8%)
MAGNETOM_ESSENZA	0 (%)	0 (%)	0 (%)
Magnetic Field			
1.5T	254 (15.1%)	36 (17.0%)	26 (12.3%)
3.0T	1427 (84.9%)	176 (83.0%)	186 (87.7%)

B value (s/mm^2)	1400 [1400, 1400]	1400 [1400, 1400]	1400 [1400, 1400]
Slice Thickness, mm	4.00 [4.00, 4.00]	4.00 [4.00, 4.00]	4.00 [4.00, 4.00]
Slice Spacing, mm	4.00 [4.00, 4.50]	4.00 [4.00, 4.50]	4.00 [4.00, 4.50]
Repetition Time, ms	3000 [2640, 4380]	2930 [2640, 4110]	2910 [2640, 4130]
Echo Time, ms	61.3 [59.7, 63.8]	61.2 [60.0, 63.7]	61.3 [59.7, 63.5]
Field of View, mm	240 [220, 250]	240 [220, 250]	240 [220, 250]
Flip Angle	90 [90, 90]	90 [90, 90]	90 [90, 90]
**T2WI**			
Slice Thickness, mm	-	-	-
Slice Spacing, mm	-	-	-
Repetition Time, ms	-	-	-
Echo Time, ms	-	-	-
Field of View, mm	-	-	-
Flip Angle	-	-	-

Table S12 Scanning protocols of the DWI/ADC images

	Overall (N=2105)	train (N=1681)	validate (N=212)	test (N=212)	*P* value
Magnetic Field					
1.5T	316 (15.0%)	254 (15.1%)	36 (17.0%)	26 (12.3%)	0.38
3.0T	1789 (85.0%)	1427 (84.9%)	176 (83.0%)	186 (87.7%)	
Manufacture					
GE MEDICAL SYSTEMS	1206 (57.3%)	959 (57.0%)	127 (59.9%)	120 (56.6%)	0.41
Philips Medical Systems	226 (10.7%)	184 (10.9%)	15 (7.1%)	27 (12.7%)	
SIEMENS	571 (27.1%)	451 (26.8%)	62 (29.2%)	58 (27.4%)	
UIH	102 (4.8%)	87 (5.2%)	8 (3.8%)	7 (3.3%)	
Model Name					
Achieva	61 (2.9%)	52 (3.1%)	5 (2.4%)	4 (1.9%)	0.87
Aera	271 (12.9%)	217 (12.9%)	32 (15.1%)	22 (10.4%)	
Amira	2 (0.1%)	1 (0.1%)	1 (0.5%)	0 (0%)	
DISCOVERY MR750	1065 (50.6%)	844 (50.2%)	112 (52.8%)	109 (51.4%)	
DISCOVERY MR750w	95 (4.5%)	75 (4.5%)	13 (6.1%)	7 (3.3%)	
Ingenia	134 (6.4%)	107 (6.4%)	8 (3.8%)	19 (9.0%)	
Ingenia CX	1 (0.0%)	1 (0.1%)	0 (0%)	0 (0%)	
Multiva	30 (1.4%)	24 (1.4%)	2 (0.9%)	4 (1.9%)	
Prisma	35 (1.7%)	29 (1.7%)	2 (0.9%)	4 (1.9%)	
SIGNA EXCITE	42 (2.0%)	36 (2.1%)	2 (0.9%)	4 (1.9%)	
Signa HDxt	3 (0.1%)	3 (0.2%)	0 (0%)	0 (0%)	
SIGNA Premier	1 (0.0%)	1 (0.1%)	0 (0%)	0 (0%)	

Skyra	60 (2.9%)	46 (2.7%)	6 (2.8%)	8 (3.8%)	
TrioTim	149 (7.1%)	114 (6.8%)	17 (8.0%)	18 (8.5%)	
uMR 790	102 (4.8%)	87 (5.2%)	8 (3.8%)	7 (3.3%)	
Verio	54 (2.6%)	44 (2.6%)	4 (1.9%)	6 (2.8%)	
B value (s/mm2)					
Median [Q1, Q3]	1400 [1400, 1400]	1400 [1400, 1400]	1400 [1400, 1400]	1400 [1400, 1400]	0.70
Repetition Time (ms)					
Median [Q1, Q3]	2930 [2640, 4370]	3000 [2640, 4380]	2930 [2640, 4110]	2910 [2640, 4130]	0.76
Echo Time (ms)					
Median [Q1, Q3]	61.3 [59.7, 63.8]	61.3 [59.7, 63.8]	61.2 [60.0, 63.7]	61.3 [59.7, 63.5]	0.97
Pixel Bandwidth (Hz)					
Median [Q1, Q3]	1950 [1570, 1950]	1950 [1630, 1950]	1950 [1540, 1950]	1950 [1610, 1950]	0.21
Reconstruction Diameter (mm)					
Median [Q1, Q3]	240 [200, 240]	240 [200, 240]	240 [200, 240]	240 [220, 240]	0.34
Slice Thickness (mm)					
Median [Q1, Q3]	4.00 [4.00, 4.00]	4.00 [4.00, 4.00]	4.00 [4.00, 4.00]	4.00 [4.00, 4.00]	0.96
Slice Spacing (mm)					
Median [Q1, Q3]	4.00 [4.00, 4.50]	4.00 [4.00, 4.50]	4.00 [4.00, 4.50]	4.00 [4.00, 4.50]	0.87
Pixel Spacing (mm)					

Median [Q1, Q3]	0.938 [0.938, 1.30]	0.938 [0.938, 1.30]	0.938 [0.938, 1.30]	0.938 [0.938, 1.30]	0.81

Categorical variables are given as numbers (percentages). Quantitative variables are given as the median [Q1, Q3] for nonnormalized data.

Table S13 Parameters for training of Model 4

Configuration		Parameter
Input to the model		
	image	label of the visible prosate cancer
Normalization of the signal intensity		
	low range	0
	high range	65535
Preprocess of the image		
	resample size	64:64:64 (z:y:x)
	crop of the image	mask of the prostate gland from Model 2
Deep learning architecture		
	model name	Unet3D
	n_filters	16
Hyper parameter		
	batch size	60
	image size	64:64:64 (z:y:x)
	num epochs	400
	learning rate	0.001
Image augmentation		

	rotation	(-10, 10)
	noise	0.0001
	affine transformation	(0, 5)
	tilt	(0, 5)
	shift	(-0.01, 0.01), (-0.01, 0.01)
Postprocess of the output		
	keep the connected domain > 500 mm^3	

Table S14 Segmentation metrics of the model

	Overall (N=2105)	train (N=1681)	validate (N=212)	test (N=212)	*P* value
DSC	0.920 [0.870, 0.940]	0.930 [0.910, 0.950]	0.810 [0.698, 0.870]	0.800 [0.615, 0.865]	<0.001
JACRD	0.850 [0.770, 0.890]	0.870 [0.830, 0.900]	0.675 [0.528, 0.770]	0.670 [0.445, 0.760]	<0.001
VS	0.990 [0.950, 0.990]	0.990 [0.980, 1.00]	0.900 [0.800, 0.950]	0.870 [0.735, 0.950]	<0.001
HD	7.40 [3.08, 13.5]	5.63 [2.50, 12.4]	9.94 [6.76, 18.0]	12.5 [7.91, 20.4]	<0.001
AD	0.110 [0.0600, 0.300]	0.0800 [0.0500, 0.180]	0.400 [0.180, 1.11]	0.490 [0.225, 1.67]	<0.001

Quantitative variables are given as the median [Q1, Q3] for nonnormalized data. DSC Dice similarity coefficient, VS volumetric similarity, HD Hausdorff distance, AD Average distance.

Table S3 Measurements of the manually labeled areas and the predicted areas (median [Q1, Q3])

	Overall (N=2105)	train (N=1681)	validate (N=212)	test (N=212)	*P* Value
Lesion number	1.00 [0, 2.00]	1.00 [0, 2.00]	1.00 [0, 2.00]	1.00 [0, 3.00]	0.56
pLesion number	1.00 [1.00, 1.00]	1.00 [1.00, 1.00]	1.00 [1.00, 1.00]	1.00 [1.00, 1.00]	0.72
Volume	1.49 [0, 11.3]	1.53 [0, 11.1]	1.36 [0, 11.8]	1.60 [0, 12.7]	0.99
pVolume	3.12 [0.864, 13.1]	3.16 [0.864, 12.9]	2.99 [0.730, 14.3]	2.99 [1.06, 13.1]	0.95
Intensity	15300 [12900, 18100]	15400 [13000, 18100]	15200 [12800, 17800]	14800 [13100, 18100]	0.78
pIntensity	16100 [13400, 18800]	16100 [13400, 18900]	16200 [13200, 18600]	15600 [13200, 18400]	0.42
DiaX	2.12 [0, 4.10]	2.11 [0, 4.10]	1.88 [0, 4.12]	2.21 [0, 4.12]	0.99
pDiaX	2.69 [1.38, 4.20]	2.64 [1.37, 4.22]	2.82 [1.39, 4.11]	2.72 [1.62, 4.18]	0.92
DiaY	1.90 [0, 3.63]	1.91 [0, 3.56]	1.89 [0, 3.73]	1.84 [0, 3.94]	0.86
pDiaY	2.32 [1.34, 3.73]	2.31 [1.34, 3.67]	2.38 [1.25, 3.90]	2.32 [1.32, 3.85]	0.81
DiaZ	1.95 [0, 3.60]	1.95 [0, 3.60]	1.70 [0, 3.58]	1.94 [0, 3.97]	0.90
pDiaZ	2.41 [1.35, 3.98]	2.41 [1.32, 3.98]	2.44 [1.31, 3.77]	2.40 [1.61, 3.98]	0.70

Table S4 Bland–Altman analysis of the measured values of prostate cancer

	RL Diameter	AP Diameter	SI Diameter	Volume	ADC value
bias	-0.464	-0.431	-0.526	-1.306	-0.464
biasUpperCI	-0.394	-0.37	-0.464	-1.053	-0.394
biasLowerCI	-0.533	-0.492	-0.588	-1.56	-0.533
biasStdDev	1.63	1.425	1.453	5.926	1.63
biasSEM	0.036	0.031	0.032	0.129	0.036
LOA_SEM	0.061	0.053	0.054	0.221	0.061
upperLOA	2.73	2.361	2.322	10.309	2.73
upperLOA_upperCI	2.85	2.465	2.428	10.742	2.85
upperLOA_lowerCI	2.611	2.257	2.216	9.876	2.611
lowerLOA	-3.657	-3.223	-3.374	-12.922	-3.657
lowerLOA_upperCI	-3.538	-3.119	-3.267	-12.489	-3.538
lowerLOA_lowerCI	-3.777	-3.327	-3.48	-13.355	-3.777
regression.fixed.slope	0.15	0.12	0.12	-0.02	0.15
regression.fixed.intercept	-0.83	-0.71	-0.82	-1.1	-0.83

LOA limits of agreement, CI confidence interval, SEM standard error of mean, ADC apparent diffusion coefficient

Table S17 Odds ratios and their significance in the models

	Value	Std. Error	*t* value	OR (95% CI)	*P* value
Age	-0.01	0.003	-3.193	0.001 (-0.032, 0.012)	0.001
PSA	0.046	0.008	5.524	1.047 (0.030, 0.063)	<0.001
PCaLocation=PZ	2.203	0.159	13.861	9.054 (0.937, 3.471)	<0.001
PCaLocation=TZ	2.001	0.135	14.792	7.396 (0.830, 3.213)	<0.001
PCaLocation=PZ+TZ	2.363	0.119	19.787	10.626 (1.150, 3.592)	<0.001
PIRADS=PIRADS_2	0.105	0.162	0.647	1.110 (-2.579, 2.752)	0.518
PIRADS=PIRADS_3	0.157	0.123	1.277	1.170 (-2.817, 2.433)	0.201
PIRADS=PIRADS_4	0.099	0.161	0.615	1.104 (-2.618, 2.625)	0.538
PIRADS=PIRADS_5	-0.156	0.17	-0.922	0.855 (-2.455, 2.797)	0.357
p_m1_volume	0.010	0.019	0.542	1.010 (-0.027, 0.048)	0.588
p_m1_z	-0.006	0.012	-0.497	0.994 (-0.031, 0.020)	0.619
p_m1_ADC	-0.002	0.000	-4.347	0.998 (-0.003, 0.000)	<0.001
p_m1_DWIsingal	0.000	0.001	0.427	1.000 (-0.001, 0.001)	0.670
p_m1_T2WIsignal	0.000	0.000	-0.576	1.000 (-0.001, 0.000)	0.565

Table S18 Selected features and their coefficients in the radiomics and deep-radiomics models

Model	Selected covariates	Coefficients
Radiomics model		
	log-sigma-5-0-mm-3D_glcm_Imc1	0.187
	original_gldm_LargeDependenceHighGrayLevelEmphasis	0.287
	original_glrlm_LongRunLowGrayLevelEmphasis	-0.123
	original_glrlm_LowGrayLevelRunEmphasis	-0.263
	original_glszm_LowGrayLevelZoneEmphasis	-0.390
	wavelet-HLH_gldm_DependenceEntropy	0.517
	wavelet-LHH_gldm_DependenceEntropy	0.357
	wavelet-LHL_gldm_DependenceEntropy	0.405
	wavelet-LLL_firstorder_Entropy	0.421
	wavelet-LLL_firstorder_Minimum	-0.162
	wavelet-LLL_firstorder_Uniformity	-0.480
	wavelet-LLL_glcm_DifferenceEntropy	0.090
	wavelet-LLL_glrlm_LongRunLowGrayLevelEmphasis	-0.169
Deep-radiomics model		
	ResNet_feature_4	0.138
	ResNet_feature_13	0.160
	ResNet_feature_56	0.278
	ResNet_feature_194	0.294
	ResNet_feature_200	0.414
	ResNet_feature_319	0.257
	ResNet_feature_363	0.285
	ResNet_feature_372	0.419
	ResNet_feature_422	-0.155

ResNet_feature_423	-0.115
ResNet_feature_471	0.127
ResNet_feature_475	0.180
ResNet_feature_487	0.362

Table S19 DeLong test of the AUCs in the training and external validation datasets

	Clinical model	PIRADS category	Radiomics model	Deep-radiomics model	Biopsy pathology
**Training dataset**					
**ISUP_1 (N=10)**					
Clinical model	NA	0.002	0.339	0.358	0.884
PIRADS category	0.002	NA	0.001	0.001	0.053
Radiomics model	0.339	0.001	NA	0.646	0.605
Deep-radiomics model	0.358	0.001	0.646	NA	0.292
Biopsy pathology	0.884	0.053	0.605	0.292	NA
**ISUP_2 (N=148)**					
Clinical model	NA	0.109	0.020	< 0.001	0.008
PIRADS category	0.109	NA	0.914	< 0.001	0.142
Radiomics model	0.020	0.914	NA	< 0.001	0.273
Deep-radiomics model	< 0.001	< 0.001	< 0.001	NA	< 0.001
Biopsy pathology	0.008	0.142	0.273	< 0.001	NA
**ISUP_3~5 (N=187)**					
Clinical model	NA	0.011	0.035	< 0.001	0.007
PIRADS category	0.011	NA	0.236	< 0.001	0.896
Radiomics model	0.035	0.236	NA	< 0.001	0.193
Deep-radiomics model	< 0.001	< 0.001	< 0.001	NA	< 0.001
Biopsy pathology	0.007	0.896	0.193	< 0.001	NA
**External validation dataset**					
**ISUP_1 (N=52)**					
Clinical model	NA	< 0.001	0.019	0.120	< 0.001
PIRADS category	< 0.001	NA	0.109	< 0.001	0.705
Radiomics model	0.019	0.109	NA	< 0.001	0.050
Deep-radiomics model	0.120	< 0.001	< 0.001	NA	< 0.001

Biopsy pathology	< 0.001	0.705	0.05	< 0.001	NA
**ISUP_2 (N=152)**					
Clinical model	NA	0.753	0.207	0.001	0.547
PIRADS category	0.753	NA	0.228	0.005	0.29
Radiomics model	0.207	0.228	NA	< 0.001	0.714
Deep-radiomics model	0.001	0.005	< 0.001	NA	< 0.001
Biopsy pathology	0.547	0.29	0.714	< 0.001	NA
**ISUP_3~5 (N=329)**					
Clinical model	NA	0.066	0.002	0.005	< 0.001
PIRADS category	0.066	NA	0.735	< 0.001	0.016
Radiomics model	0.002	0.735	NA	< 0.001	0.111
Deep-radiomics model	0.005	< 0.001	< 0.001	NA	< 0.001
Biopsy pathology	< 0.001	0.016	0.111	< 0.001	NA

Table S20 DeLong test of the AUCs among different hospitals in the external validation dataset

	ISUP_2 (N=152)			ISUP_3~5 (N=329)		
	Hospital_2	Hospital_3	Hospital_4	Hospital_2	Hospital_3	Hospital_4
**Clinical model**						
Hospital_2	NA	0.686	0.848	NA	0.399	0.981
Hospital_3	0.686	NA	0.074	0.399	NA	0.008
Hospital_4	0.848	0.074	NA	0.981	0.008	NA
**PIRADS category**						
Hospital_2	NA	0.822	0.457	NA	0.655	0.413
Hospital_3	0.822	NA	0.148	0.655	NA	0.278
Hospital_4	0.457	0.148	NA	0.413	0.278	NA
**Radiomics model**						
Hospital_2	NA	0.638	0.784	NA	0.421	0.664
Hospital_3	0.638	NA	0.554	0.421	NA	0.239
Hospital_4	0.784	0.554	NA	0.664	0.239	NA
**Deep-radiomics model**						
Hospital_2	NA	0.970	0.826	NA	0.665	0.840
Hospital_3	0.970	NA	0.602	0.665	NA	0.464
Hospital_4	0.826	0.602	NA	0.840	0.464	NA
**Biopsy pathology**						
Hospital_2	NA	0.559	0.796	NA	0.614	0.919
Hospital_3	0.559	NA	0.021	0.614	NA	0.082

Hospital_4	0.796	0.021	NA	0.919	0.082	NA

Supplementary Figures

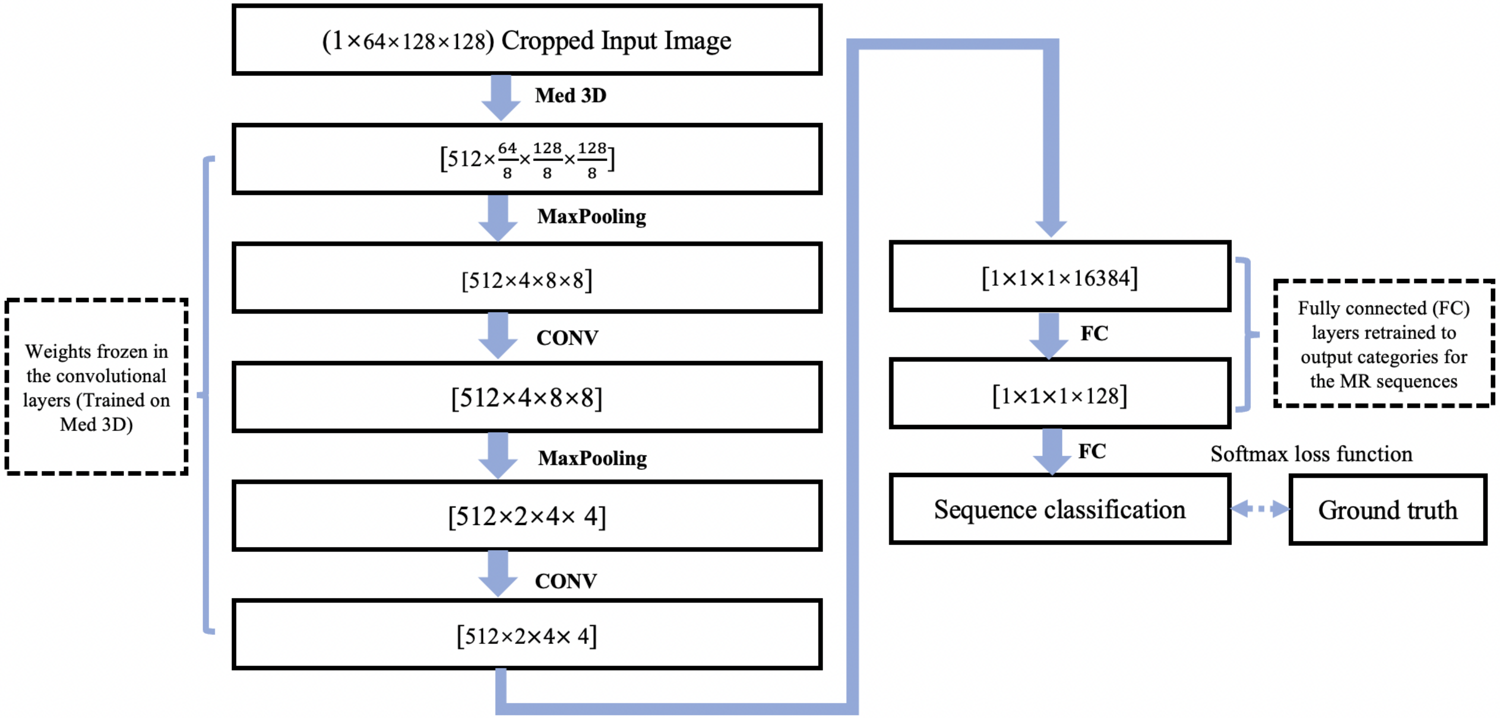

Figure S1 The modified Med3D network. 3D three-dimensional, CONV Convolution, FC Fully connected.

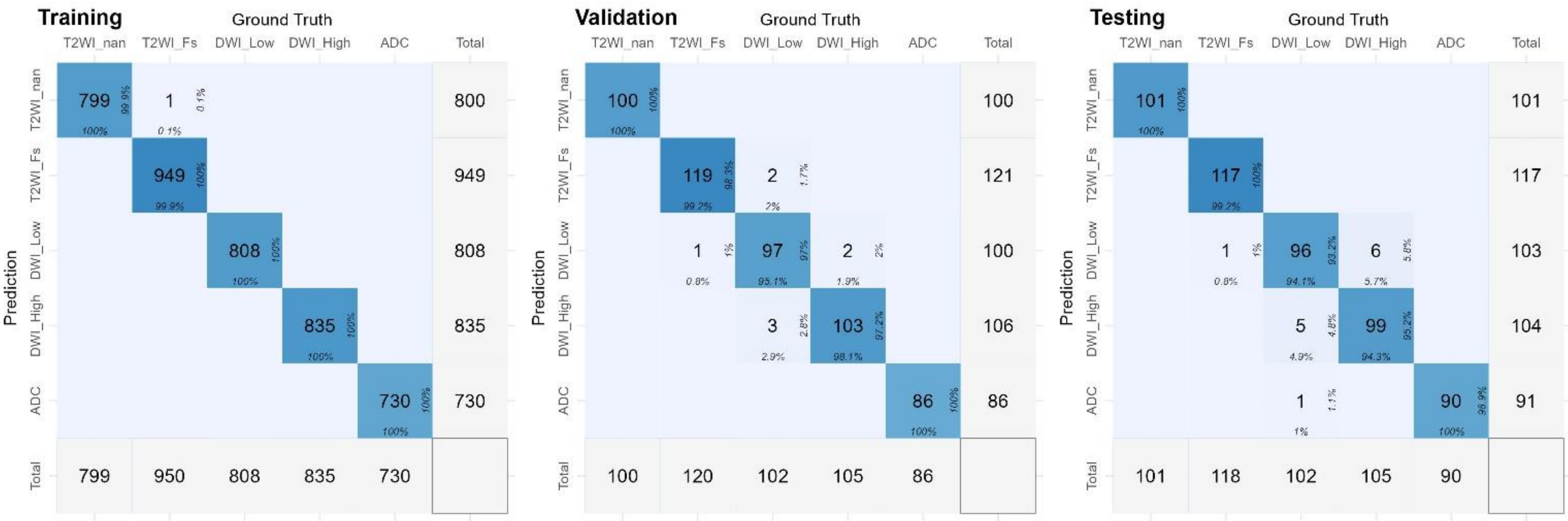

Figure S2 Confusion matrix of the prediction results in the training, validation, and testing datasets. The number in the middle of each tile is the counted number of images. The percentage number at the bottom of each tile is the column percentage. The percentage number on the right side of each tile is the row percentage. The color intensity is based on the counts. T2WI T2-weighted imaging, Fs fat saturation, DWI diffusion-weighted imaging, ADC apparent diffusion coefficient.

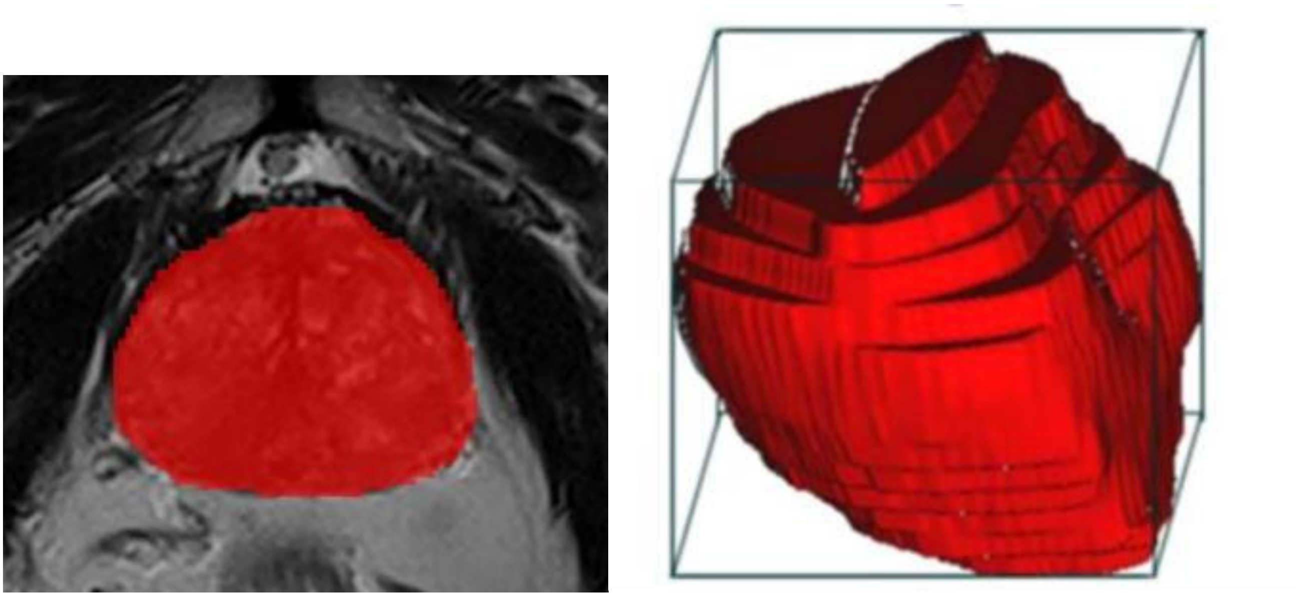

Figure S3 Whole prostate segmentation and the algorithm rule of the minimum volume bounding box.

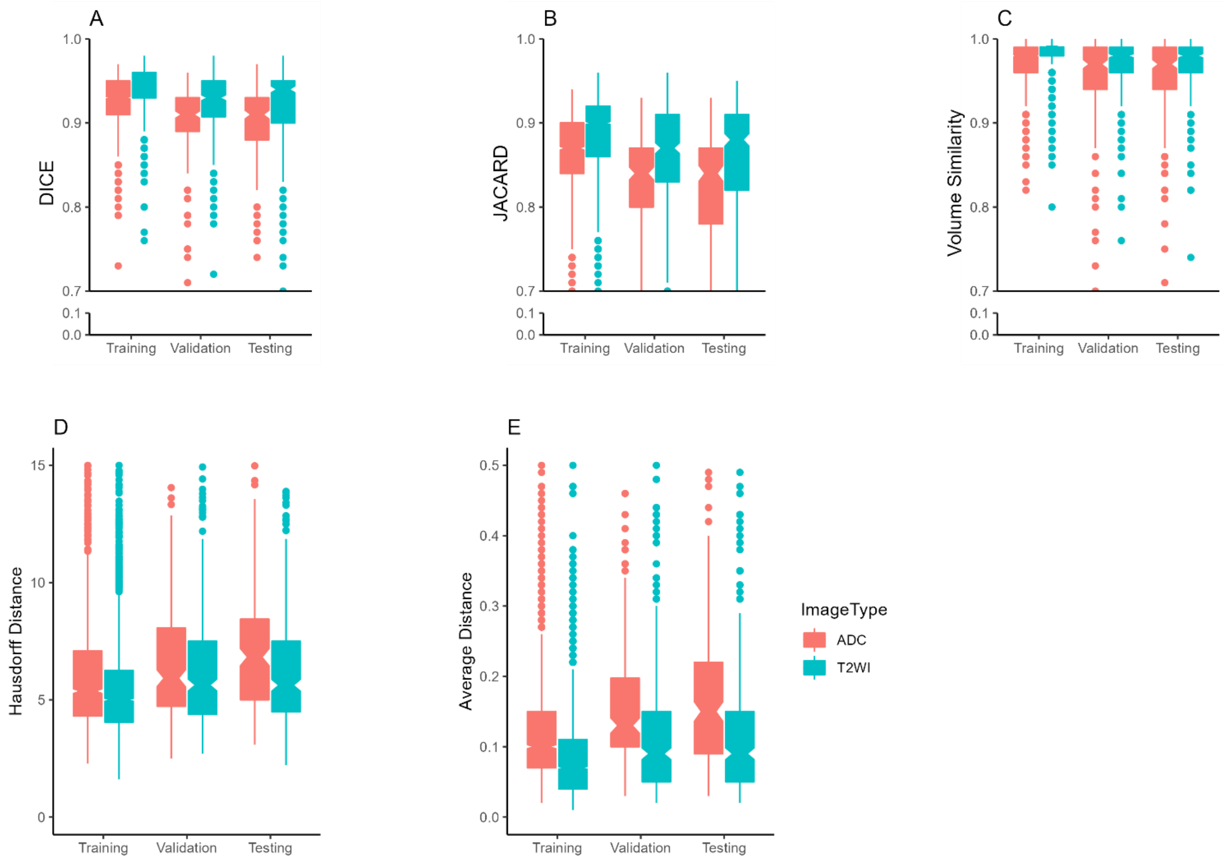

Figure S4 The DSC, Jaccard index, VS, HD, and AD in different datasets. The metrics of the T2WI were superior to those of the ADC map in all datasets (all P< 0.001). DSC Dice similarity coefficient, VS volumetric similarity, HD Hausdorff distance, and AD Average distance. T2WI T2-weighted imaging, ADC apparent diffusion coefficient.

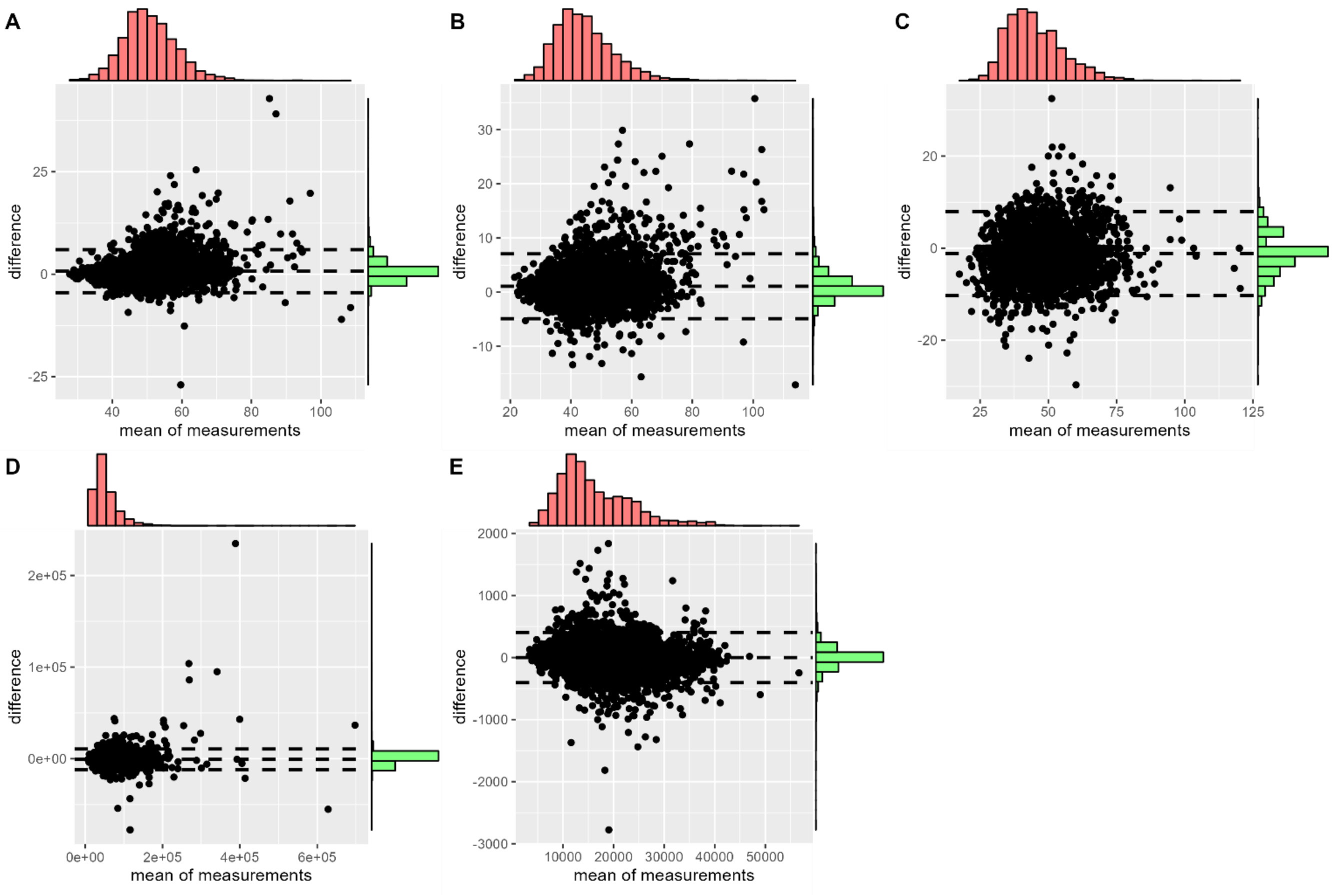

Figure S5 Bland–Altman analysis of the values of the RL diameter (A), AP diameter (B), SI diameter (C), volume (D), and signal intensity (E) of the manual label and the predicted label of the prostate gland. RL right and left, AP anterior and posterior, SI superior and inferior.

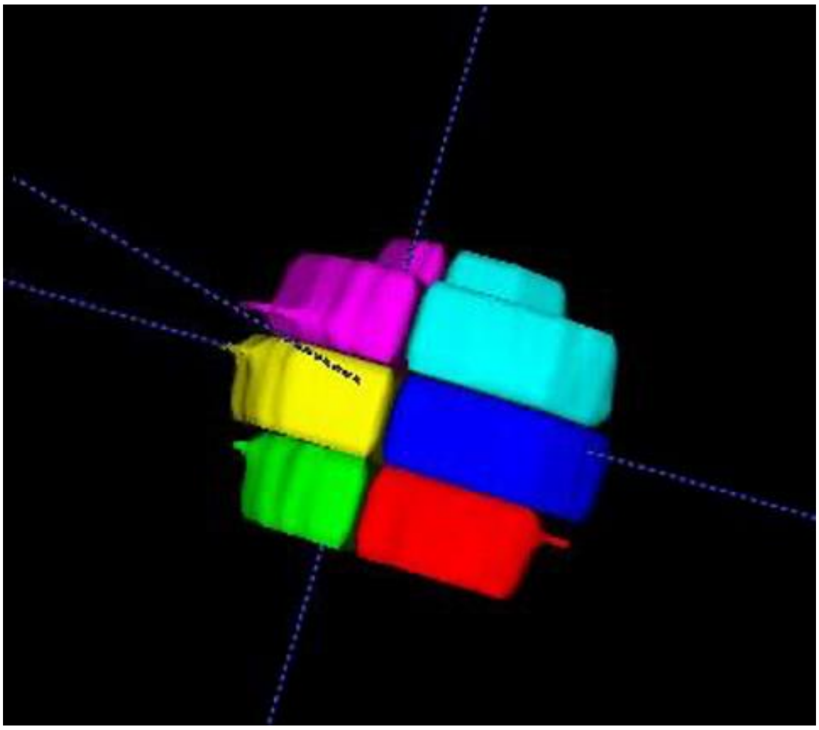

Figure S6 Sextant locations.

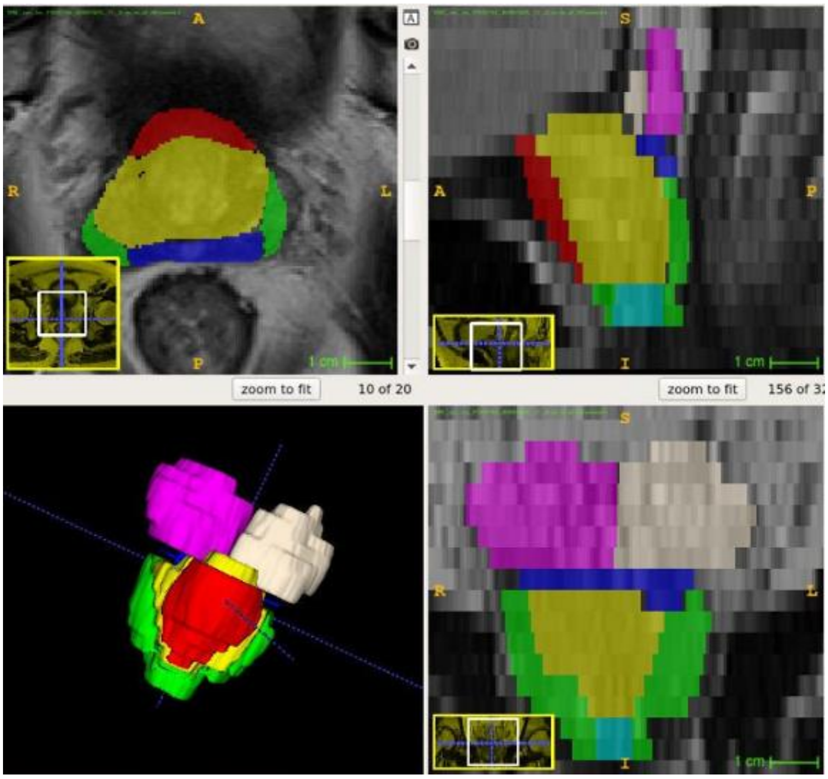

Figure S7 Anatomic zone locations.

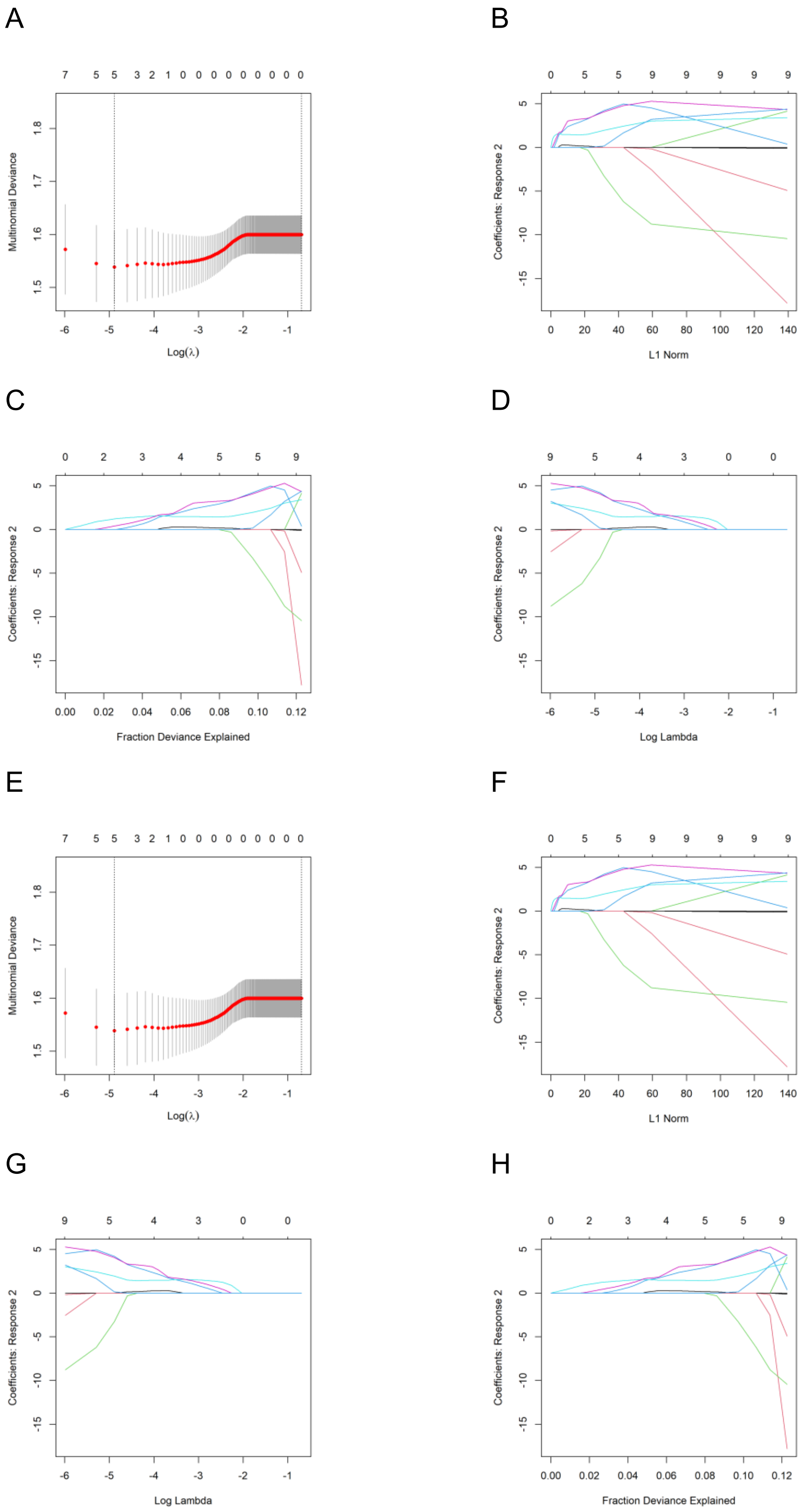
A
Multinomial Deviance
Log(λ)
B
Coefficients: Response 2
L1 Norm
C
Coefficients: Response 2
Fraction Deviance Explained
D
Coefficients: Response 2
Log Lambda
E
Multinomial Deviance
Log(λ)
F
Coefficients: Response 2
L1 Norm
G
Coefficients: Response 2
Log Lambda
H
Coefficients: Response 2
Fraction Deviance Explained

Figure S8 Development of the radiomics and deep-radiomics LASSO classification. A to D how the development of the radiomics model. E to H show the development of the deep-radiomics model. A and E provide visual representations of the cross-validated performance of the LASSO regression model across different values of the regularization parameter lambda. The x-axis represents the logarithm (base 10) of the lambda values used in the cross-validation process. The y-axis represents the multinomial deviance across all folds in the cross-validation process. The vertical lines indicate different lambda values. These lines represent the cross-validated error for each lambda value. The dotted vertical line at a specific point on the x-axis represents the lambda value that gives the minimum cross-validated error. This is considered the optimal lambda for the Lasso model. The red dots compose the performance curve, which helps us understand the trade-off between model complexity and performance. The cross-validation process helps in selecting an optimal lambda value for the LASSO regression model, which in turn helps prevent overfitting and improves the model's ability to generalize to new data.

The other plots provide detailed insights into how the coefficient for the predictor variable is influenced by different aspects of the LASSO regression model, including the L1 norm (B and F), the logarithm of the regularization parameter (lambda) (Figure C and G), and the fraction of deviance explained (D and H) by the model.

References

1. Sun Z, Wu P, Cui Y, et al. Deep-Learning Models for Detection and Localization of Visible Clinically Significant Prostate Cancer on Multi-Parametric MRI. J Magn Reson Imaging 2023;58:1067-1081. DOI: 10.1002/jmri.28608.

2. Sun Z, Wang K, Wu C, et al. Using an artificial intelligence model to detect and localize visible clinically significant prostate cancer in prostate magnetic resonance imaging: a multicenter external validation study. Quant Imaging Med Surg 2024;14:43-60. DOI: 10.21037/qims-23-791.

3. Yin X, Wang K, Wang L, et al. Algorithms for classification of sequences and segmentation of prostate gland: an external validation study. Abdom Radiol (NY) 2024;49:1275-1287. DOI: 10.1007/s00261-024-04241-8.

4. Zhang J, Yin X, Wang K, et al. External validation of AI for detecting clinically significant prostate cancer using biparametric MRI. Abdom Radiol (NY) 2024. DOI: 10.1007/s00261-024-04560-w.

5. Robin X, Turck N, Hainard A, et al. pROC: an open-source package for R and S+ to analyze and compare ROC curves. BMC Bioinformatics 2011;12:77. DOI: 10.1186/1471-2105-12-77.

6. Hand DJ, Till RJ. A Simple Generalisation of the Area Under the ROC Curve for Multiple Class Classification Problems. Machine Learning 2001;45:171-186. DOI: 10.1023/A:1010920819831.
